# Identification of target-binding peptide motifs by high-throughput sequencing of phage-selected peptides

**DOI:** 10.1093/nar/gku940

**Published:** 2014-10-27

**Authors:** Inmaculada Rentero Rebollo, Michal Sabisz, Vanessa Baeriswyl, Christian Heinis

**Affiliations:** Institute of Chemical Sciences and Engineering, Ecole Polytechnique Fédérale de Lausanne, CH-1015 Lausanne, Switzerland

## Abstract

High-throughput sequencing was previously applied to phage-selected peptides in order to gain insight into the abundance and diversity of isolated peptides. Herein we developed a procedure to efficiently compare the sequences of large numbers of phage-selected peptides for the purpose of identifying target-binding peptide motifs. We applied the procedure to analyze bicyclic peptides isolated against five different protein targets: sortase A, urokinase-type plasminogen activator, coagulation factor XII, plasma kallikrein and streptavidin. We optimized sequence data filters to reduce biases originating from the sequencing method and developed sequence correction algorithms to prevent identification of false consensus motifs. With our strategy, we were able to identify rare target-binding peptide motifs, as well as to define more precisely consensus sequences and sub-groups of consensus sequences. This information is valuable to choose peptide leads for drug development and it facilitates identification of epitopes. We furthermore show that binding motifs can be identified after a single round of phage selection. Such a selection regimen reduces propagation-related bias and may facilitate application of phage display in non-specialized laboratories, as procedures such as bacterial infection, phage propagation and purification are not required.

## INTRODUCTION

Phage display of peptides is widely used for the development of peptide ligands and for epitope mapping (1,2). The procedure involves 2–4 iterative rounds of affinity selection and phage amplification followed by an optional ELISA-based screen and sequencing of several dozens of positive clones. A panel of peptides is synthesized and their binding to the protein target or biological activity tested. An important step in the phage selection of peptides is the comparison of sequences and the identification of consensus motifs. Consensus sequences can provide valuable information about the binding site of peptides. Peptides sharing the same consensus motif likely bind to the same surface region of the target protein and form similar molecular interactions. Multiple different consensus sequences indicate that peptides bind with different interaction modes to the same or different surface regions. Selections with peptide libraries often yield only one consensus sequence or at maximum a few different ones. In many phage selections with peptide libraries, no consensus sequences are reported at all. If isolated ligands are to be used as leads in drug development, multiple consensus sequences are desired as parallel development of several peptide leads increases the success rate of the development program. For example, peptides of one consensus sequence might share unfavorable properties such as poor solubility or low proteolytic stability, hindering their further development.

The sequences of phage-selected peptides are typically obtained by Sanger sequencing. Our laboratory, for example, is routinely sequencing a half or a whole 96-well plate of clones isolated after 2–3 rounds of phage panning. Sequence similarities among peptides are identified by manual comparison of the sequences and highlighted by coloring amino acids of aligned peptides or by representation as so-called logos. In recent years, high-throughput sequencing (HTS) methods have been applied for the analysis of ligands isolated from DNA-encoded chemical libraries (3,4), or antibodies (5,6), protein domains (7–9) and peptides (10–16) isolated from phage display or mRNA display libraries. Most of the sequencing work was done using Roche's 454 sequencing technology (earlier work) (3,6,7,10), the Illumina platform (4,5,8,9,11–16) or an Ion Torrent sequencer (12). The vast sequence data gave valuable information about diversity and abundance of isolated clones, as well as allowed monitoring of these parameters during the different iterative rounds of selection and amplification. In selections of peptide ligands, the sequencing data was analyzed primarily by ranking the peptides according to their abundance, and the most frequent peptides were characterized. ‘t Hoen *et al.* (16) and Olson *et al.* (9) showed that peptide ligands can be identified in a single round of selection. In order to distinguish functional clones over background, Olsen *et al.* subjected each clone in > 1000 copies to the selection (input) and identified potential binding sequences from the 10 most abundant peptides. Herein, we proposed to analyze HTS data of phage-selected peptides not only based on abundance, but also based on sequence homology. We expected that sequencing and comparison of ten-thousends of peptides could allow a finer discrimination of consensus sequence sub-groups. Extensive sequence homology information could provide information about binding interactions and the importance of specific residues for the binding.

Powerful tools to compare extensive sequence data and to identify multiple different consensus sequences within large datasets of sequences have been developed. The algorithms of Multiple Em for Motif Elicitation (15), MUltiple Specificity IdentifIer (17) and Gibbs Cluster (18) can process large numbers of sequences and group them in clusters of similar peptides. The three tools unfortunately do not provide information about frequencies and nucleotide sequences in the analysis result. Derda *et al.* developed MatLab-based software for the analysis of phage-selected peptides sequenced by the Illumina platform (11). The tool tailored for the commercial Ph.D.^TM^ -12 Phage Display Library (New England Biolabs) provides information about sequence abundance and DNA sequences but it does not include a function for automated identification of sequence homologies.

In this work, we conceived a strategy to identify target-binding peptide motifs by HTS and sequence comparison. We developed a procedure and software for vast data processing, sequence quality filtering and homology finding. We applied it to bicyclic peptides that were isolated against five different protein targets. The tools allowed identification of numerous sub-families of consensus sequences. We show that target-binding peptide motifs can be identified even after only one round of affinity selection.

## MATERIALS AND METHODS

### Phage selection

Libraries A, B, 3×3 and 4×4 were previously described (19,20). In these libraries, peptides are displayed on around five copies of the phage coat protein pIII. Libraries A and B contain peptides of the format ACX_m_CX_n_CG (C = cysteine, X = any amino acid). In library A, the combinations of ‘m’ and ‘n’ are 3/4, 4/3, 4/4, 3/5 and 5/3; in library B they are 3/6, 6/3, 4/5 and 5/4. Library 3×3 contains peptides of the format XCX_3_CX_3_CX. Library 4×4 contains peptides of the format XCX_4_CX_4_CX. Random positions are coded by NNK codons. Phage production, reaction of cysteines with chemical linker to generate bicyclic peptides on phage and phage panning against the different targets were performed as described before (19–21). The vector for *Staphylococcus aureus* sortase A expression pHTT14 (22) was kindly provided by Prof. O. Schneewind. Sortase A was expressed in *Escherichia coli* (amino acid 26–206, polyhistidine tag at N-terminus) and purified by nickel affinity chromatography followed by size exclusion chromatography. Human urokinase-type plasminogen activator N322Q was expressed in mammalian cells, activated and purified as described before (23). Human coagulation factor XIIa (β-form) and human plasma kallikrein were purchased from Molecular Innovations (Novi, MI, USA). The proteins were biotinylated and immobilized on streptavidin magnetic beads (Dynabeads M-280, Life Technologies, Carlsbad, CA, USA). For SA selections, the commercial SA magnetic beads were readily used.

### Sample preparation for HTS

Phage vector was extracted from TG1 *E. coli* bacteria that were stored as glycerol stocks after infection with phage isolated after one round of selection. The DNA was isolated with a commercial plasmid purification kit (NucleoSpin Plasmid; Macherey-Nagel, Düren, Germany). Hundred nanogram phage vector DNA was amplified by PCR using primers containing adapter sequences and barcodes (primer sequences are provided in Supplementary Table S1). The PCR reaction in a volume of 50 μL contained final concentrations of 250 μM dNTP, 500 nM primer, 1 unit Taq polymerase and standard buffer (75 mM Tris-HCl, 20 mM (NH_4_)_2_SO_4_, 2 mM MgCl_2_, 0.01% Tween 20). Twenty-five PCR cycles (30 s 95°C, 30 s 55°C, 30 s 72°C) were performed but resulted in formation of DNA heteroduplexes and only about 30% of all sequences could be read. Reduction of the number of PCR cycles to 13 solved this problem. PCR products were purified from a 2.5% agarose gel (UltraPure agarose, Invitrogen, Carlsbad, CA, USA) using a commercial agarose gel purification kit (NucleoSpin Gel and PCR Clean-up; Macherey-Nagel). The concentration of DNA was determined using a High Sensitivity DNA Assay Kit (Agilent, Santa Clara, CA, USA), following the manufacturer's protocol. Ion Torrent sequencing was performed by the Lausanne Center of Genomic Technologies (University of Lausanne, Switzerland) or the Centre for Research in Agricultural Genomics (Barcelona, Spain) on a Ion Personal Genome Machine (PGM^TM^) Sequencer. The procedure involved ligating the DNA fragments onto Ion Sphere Particles (ISPs), amplifying them by emulsion PCR, enriching the templated ISPs, loading onto an Ion Torrent 316^TM^ chip and sequencing.

### Analysis

MatLab scripts were developed for the analysis of HTS data (all scripts and descriptions can be found in the Supplementary Data). A first script, *Step1.m*, sorts the reads according to the specified barcodes and distributes them to separate files. Reads with mutations, insertions or deletions in barcodes were discarded unless specified. A second script, *Step2.m*, removes low-quality reads, translates the sequences, sorts them by abundance and optionally corrects sequencing errors. Reads having more than three bases with quality score lower than Q18 were not considered, unless specified otherwise. Sequences differing in one or two bases from an abundant sequence were corrected as the small differences likely origin from sequencing errors. MatLab scripts *LoopLengths.m*, *Clustering.m*, *FindSeq.m* and *CommonSeq.m* were used for the comparison and analysis of peptide sequences. Script *LoopLengths.m* separates the sequences into different files according to the number of cysteine residues and the number of amino acids between them. Script *Clustering.m* compares a chosen number of sequences, groups them into families that share high sequence similarity and optionally generates sequence logos for each group. Script *FindSeq.m* searches the dataset for all peptide sequences containing a specified motif. Script *CommonSeq.m* compares up to three different datasets and distributes common and exclusive sequences in different files.

## RESULTS

### Phage selection and HTS

**Figure 1.**
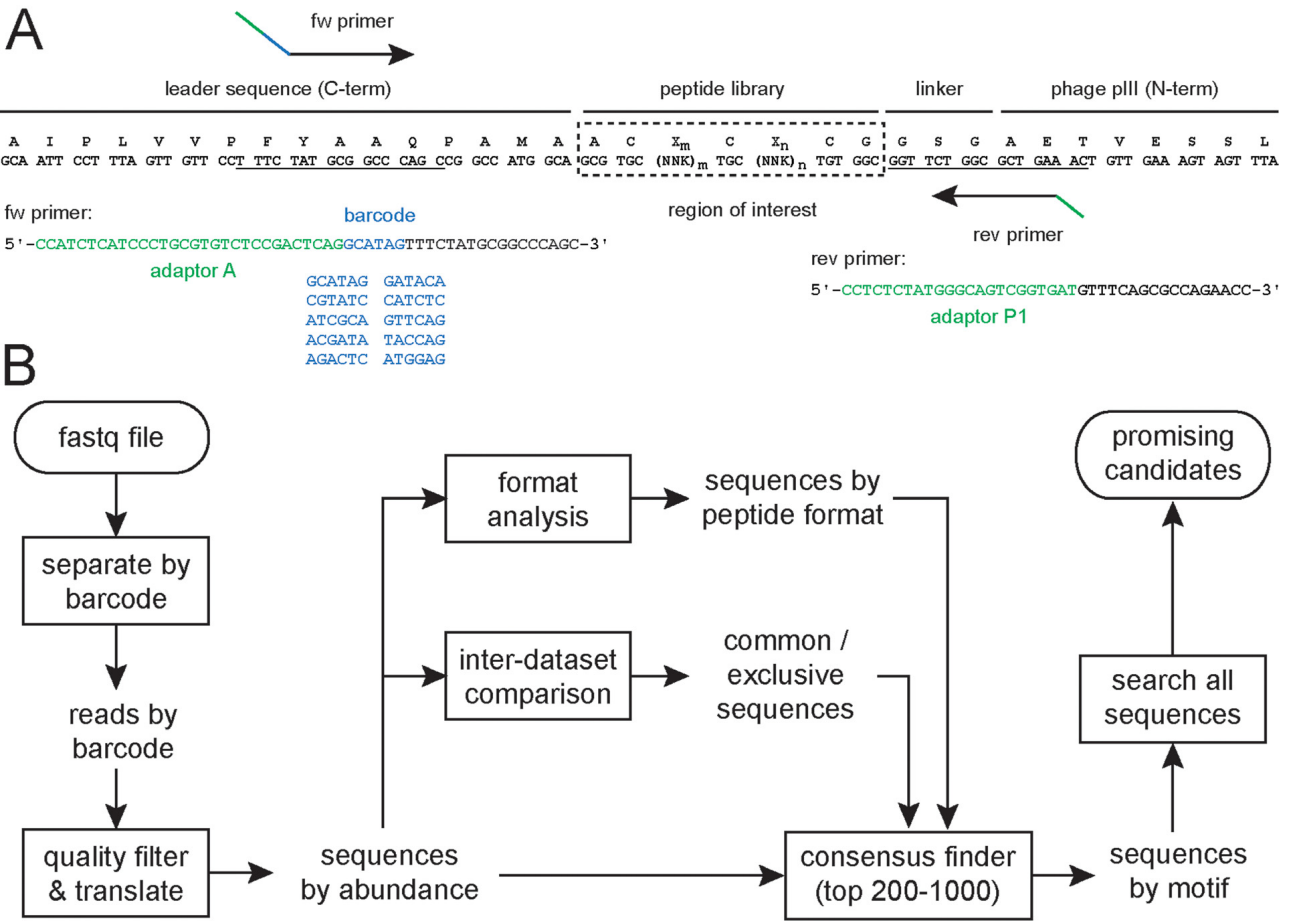
HTS and sequence analysis strategy. (**A**) Primer design for Ion Torrent sequencing of bicyclic peptide libraries (Library A and Library B). (**B**) Procedure for the analysis of sequencing data applying MatLab scripts. First, reads are separated into several files according to their barcode. Second, low-quality sequences are removed from the dataset, and remaining sequences are translated and sorted by abundance. At this point, two optional steps are performed: distribution of the isolated peptide sequences based on the format (e.g. peptide ring size in the case of bicyclic peptides) and comparison of two different datasets. Then, the sequences of the most abundant peptides (e.g. top 200) are compared and clustered in consensus groups or sub-families of consensus groups, allowing the identification of specific motifs. Finally, the entire pool of sequences is searched for other less abundant sequences sharing such motifs in order to identify promising candidates.

Bicyclic peptide phage libraries were generated by displaying linear peptides of the format X_l_CX_m_CX_n_CX_o_ (C = cysteine; X = any amino acid, l, m, n, o = number of random amino acids) on filamentous phage and subsequent chemical cyclization of the peptides with tris-(bromomethyl)benzene (TBMB) (24). The libraries were panned against the five targets sortase A from *S. aureus* (SrtA), human urokinase-type plasminogen activator (uPA), activated human coagulation factor XII (FXIIa), human plasma kallikrein (PK) and streptavidin (SA). Bicyclic peptide libraries were previously screened against four of these targets (uPA, FXIIa, PK, SA) and had yielded binders with micromolar to picomolar dissociation constants (19–21,25). In these previous selections, consensus sequences were identified by sequencing around 100–300 clones per target after 2–3 iterative selection rounds. Against the bacterial target SrtA of *S. aureus*, bicyclic peptides were not developed so far. Isolated peptides were analyzed after a single round of phage selection instead of after 2–3 iterative rounds, as usually done. A single round of selection minimizes out-competition of weaker binders by stronger ones. In this way, a maximal number of target-binding peptide motifs was expected to be identified. A risk of a single round of selection was that binders were not sufficiently enriched over non-binders, making the identification of consensus sequences more difficult. Bicyclic peptide libraries with different format (ring sizes of 3–6 amino acids), different complexity (10^7^ to 4×10^9^ different clones) and different representation of individual phage clones (ranging from 2 to 1000 copies per clone) were applied as shown in Table 1. In some selections, phage clones were represented in high copy numbers to facilitate enrichment of individual clones over non-specifically selected ‘background’ peptides. This was expected to facilitate analysis of data and identification of consensus sequences. After one round of phage selection, phage DNA was sequenced on an Ion PGM^TM^ Sequencer instrument using an Ion 316^TM^ Chip, yielding a maximum of 5×10^6^ reads per chip. This number was exceeding by far the number of phage isolated in the phage selections, ranging from 4×10^2^ to 3×10^4^ (Table 1). It even allowed sequencing phage from multiple selections on a single chip. DNA of selected phage was isolated from bacterial cells and amplified by PCR using suitable primers as shown in Figure 1A and Supplementary Table S1. A six-letter barcode was included in the forward primers right after the adaptor sequence to allow multiplexing of up to 10 different phage selections on a single chip. Samples run on an Ion 316^TM^ Chip yielded more than a million reads and thus more than 100 000 sequences per phage selection (Table 1).

**Table 1.** Summary of the protein targets and peptide phage display libraries

Target	Library^a^	Library diversity^b^	Phage input^c^	Phage output^c^	Ion Torrent reads	Total sequences^d^	Different sequences^e^	% top 200^f^
SrtA	Library A	5 × 10^8^	3 × 10^10^	8 × 10^3^	1.8 × 10^5^	5.1 × 10^4^	2.8 × 10^3^	26%
SrtA	Library B	1 × 10^7^	2 × 10^10^	1 × 10^4^	2.4 × 10^5^	6.8 × 10^4^	1.4 × 10^3^	75%
uPA	Library B	1 × 10^7^	9 × 10^9^	3 × 10^4^	3.4 × 10^5^	1.1 × 10^5^	3.1 × 10^3^	56%
FXIIa	4×4	7 × 10^8^	5 × 10^10^	1 × 10^4^	4.1 × 10^5^	1.7 × 10^5^	7.9 × 10^3^	15%
PK	3×3, 4×4	1 × 10^9^	2 × 10^9^	2 × 10^3^	1.8 × 10^5^	7.5 × 10^4^	1.4 × 10^3^	40%
SA	3×3, 4×4	1 × 10^9^	2 × 10^9^	4 × 10^2^	1.1 × 10^5^	6.1 × 10^4^	3.4 × 10^2^	84%

^a^Libraries are named according to reference (24). Library A contains 3×4, 4×3, 4×4, 3×5, 5×3 peptides, library B contains 3×6, 6×3, 4×5, 5×4 peptides.

^b^Number of transformants.

^c^Transducing units (t.u.).

^d^Total number of sequences after quality filter.

^e^Estimated number of different sequences.

^f^Percentage of the population corresponding to the top 200 clones.

### Data processing and analysis

We developed MatLab software for the processing and analysis of sequence data as outlined in the flow diagram shown in Figure 1B. Several of the applied procedures such as sorting of sequences, quality filtering, abundance ranking and translation were based on scripts developed by Derda and co-workers (11). In a first step, sequences provided by the Ion Torrent sequencer in fastq format (26) were distributed into different files according to their barcode to separate peptides from different phage selections. For barcodes having a single base mutation, deletion or insertion, a correction function was developed but it proved to rescue only a small fraction of peptides and was not further used (described in Supplementary Data and Supplementary Figure S1). In a second step, low-quality sequences were removed from the dataset, and identical sequences sorted by their abundance. In the same step, the DNA sequences were translated into amino acid sequences. The software allows specifying the start and end of the region to be evaluated, so that it can be applied to any peptide library, regardless of the length of the random sequence and the flanking residues. In a third step, peptides were optionally sorted according to their format (i.e. number of cysteines and number of residues between them) or based on inter-dataset comparisons (e.g. peptides isolated in two independent phage selections). In a fourth step, peptides were pairwise compared to find consensus sequences and to identify target-binding motifs. In a fifth and last step, identified peptide motifs were used to search the entire dataset for more related sequences. In all processes, information about peptide abundance and nucleotide sequence is displayed. All scripts and descriptions are available in the Supplementary Data. The scripts can be used for the analysis of any files in fastq format and thus also for data sequenced with other technology platforms such as Illumina.

### Reducing bias by optimizing quality parameters

**Figure 2.**
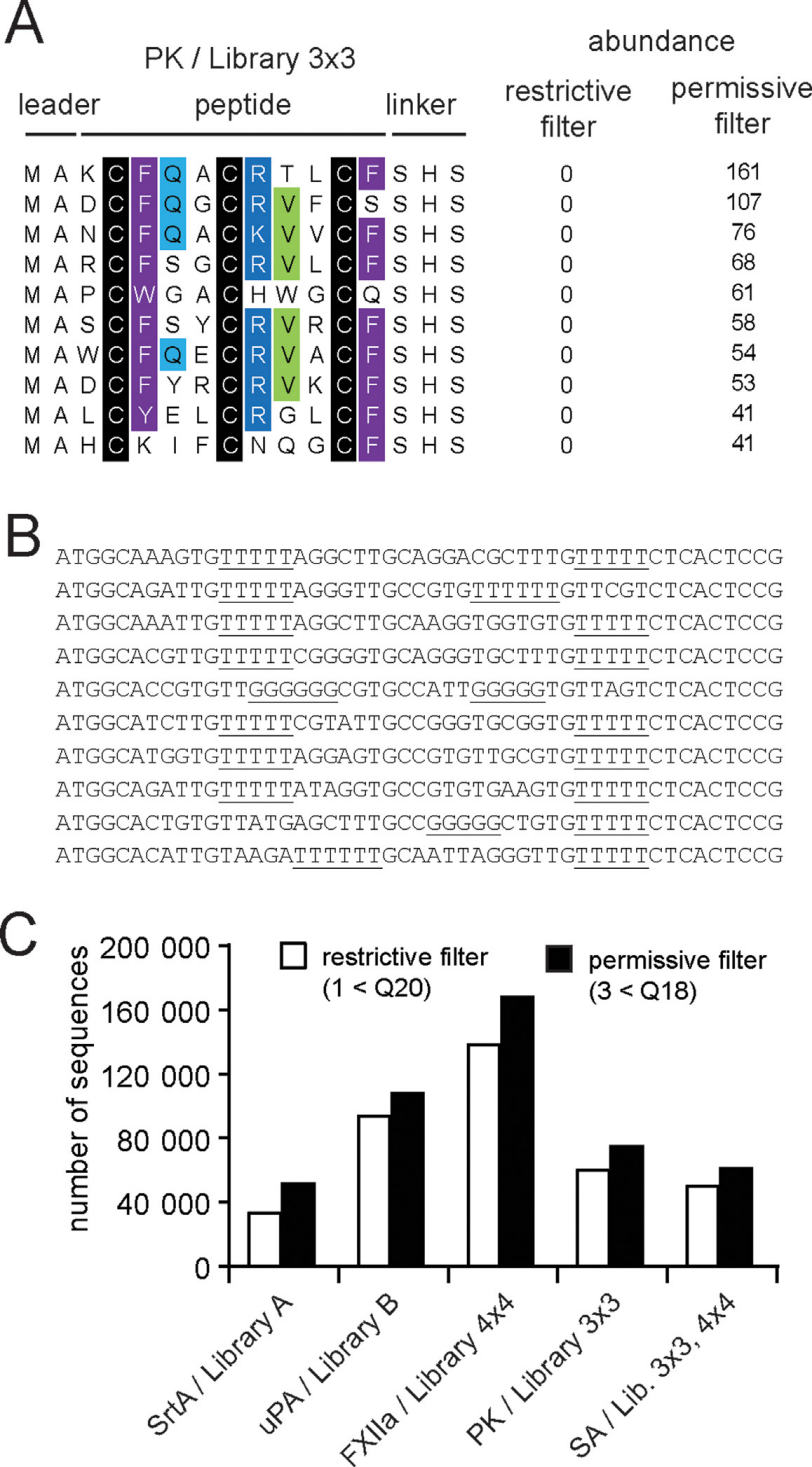
Application of an optimal sequencing quality filter. Comparison between permissive (a maximum of three bases with quality value lower than Q18 are allowed) and restrictive (a maximum one base with quality value lower than Q20 is allowed) filtering parameters. (**A**) Example of peptides rescued by applying a less restrictive quality filter to the selection against PK. The rescued peptides with the highest abundance are indicated (top 10). (**B**) DNA sequence of rescued peptides. The homopolymers in the DNA sequences are underlined. (**C**) Effect on the number of reads passing the different filtering parameters.

A critical step in the data processing is the filtering of sequencing data based on quality criteria. Ion Torrent is prone to over-calling or under-calling the length of homopolymeric regions, leading to insertion/deletion (indel) errors (27). The confidence of each sequenced nucleotide (Q-value) is provided in the fastq file with a single-character Phred-based quality score (26), assigned by the PGM base-caller. This information can be used to remove sequences containing low confidence basecalls prior to sequence analysis. Application of too strict quality filters, however, can lead to a bias against homopolymer-containing sequences. Different quality filter stringencies were tested and an optimal one chosen. A filter allowing a maximum of 3 nucleotides having a quality score below ‘Q18’ was found to be optimal for all datasets. The importance of optimal quality filtering is illustrated in Figure 2 in which different quality filters were applied to peptides isolated from the 4×4 library against PK (Figure 2A): a ‘permissive’ filter, in which reads containing 3 nucleotides below quality score ‘Q18’ were discarded, and a ‘restrictive’ filter, where only 1 nucleotide below quality score ‘Q20’ was allowed. Although the difference in the total number of reads passing each filter was minimal (Figure 2C), certain peptide sequences were completely lost when using the restrictive filter (Figure 2A). DNA of such peptides contained a tetra-thymine homopolymer (TGT-TTT; encoding Cys-Phe) as well as a penta-thymine homopolymer (TGT-TTT-TCT; encoding Cys-Phe-Ser) (Figure 2B). We observed similar biases in all datasets. In order to reduce the bias against sequences containing long homopolymers, the less strict quality filter with a maximum of 3 nucleotides under the quality score ‘Q18’ was applied.

### Diversity of phage-selected peptides

**Figure 3.**
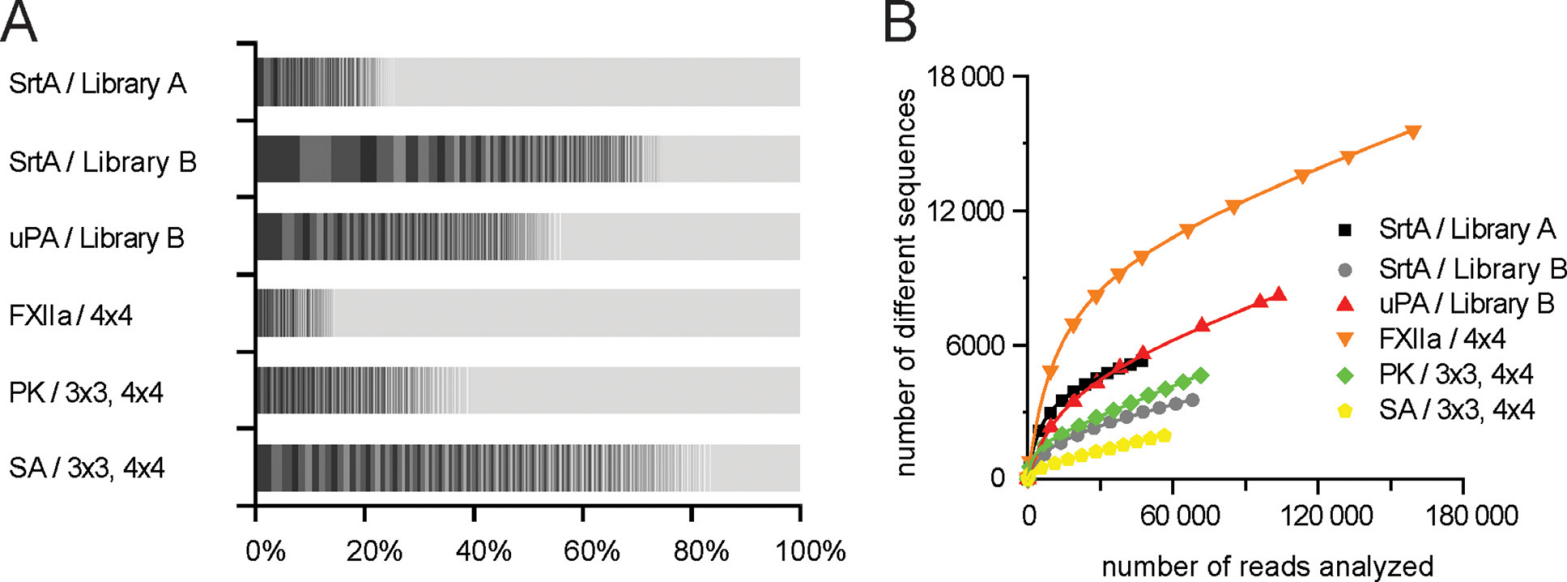
Diversity of peptides isolated after one round of phage selection. (**A**) The abundance of the 200 peptides that were most frequently found is indicated in percent of the whole population of sequenced clones (indicated in blocks colored in different grayscales). (**B**) Number of different sequences found when increasing numbers of reads were analyzed. Saturation plots were used for the calculation of the total number of different sequences.

The copy number of the most abundant peptides varied strongly among different selections. In some selections, the 200 most frequently found peptides represented more than 80% of the sequenced clones, while in other selections they formed a fraction of less than 20% (Figure 3A). We plotted the number of different peptide sequences against the number of analyzed reads to extrapolate the absolute number of different peptide sequences found in each selection. We expected that the number of different sequences converges to a maximal value at larger numbers of analyzed peptides and fits to Equation (1) where *a* is the total number of different peptide sequences in the dataset and *k* is a constant that depends on the abundance distribution of the sample. Equation (1) was found to be suitable for fitting simulated datasets containing (i) different numbers of peptides and (ii) different peptide abundance distributions (Supplementary Data and Supplementary Figures S2 and S3).

(1) \begin{equation*} y = a\left( {1 - e^{ - \frac{x}{k}} } \right) \end{equation*}

The number of different sequences increased linearly at larger numbers of sequences analyzed and did not converge to a maximal value, as well as did not fit to Equation (1). The linear increase was due to sequencing errors, which were directly proportional to the number of sequences. Taking this phenomenon into account, we fitted the data to Equation (2) where *a* and *k* are again the total number of different peptide sequences in the dataset and a constant that depends on the abundance distribution of the sample, respectively, and *b* is the average error rate of the population. Equation (2) was also verified with simulated datasets containing (i) different number of peptides, (ii) different abundance distributions and (iii) different error rates (Supplementary Data and Supplementary Figure S4).

(2) \begin{equation*} y = a\left( {1 - e^{ - \frac{x}{k}} } \right) + bx \end{equation*}

Data of all selections was fitting well to Equation (2) (Figure 3B). The linear coefficient *b* was similar in datasets of all selections. Experimental datasets of this study contained a significant percentage of sequencing errors estimated to be between 2.8% and 5.1%. The number of different sequences calculated for the various selections ranged between 340 and 8000 and was hence consistent with the number of isolated phage (Table 1). The different peptides isolated in selections could thus essentially all be identified by analysing around 100 000 reads.

After one round of phage selection, we expected that propagation advantages of specific clones would not have a large impact on the selection results. To evaluate the extent of the propagation-related bias after one round of selection, phage of library A and B were produced and bacterial cells infected without affinity selection. The copy number of individual clones increased only marginally and the most abundant clones represented in both cases less than 0.02% of the population (Supplementary Figure S5) and were not found after selection.

### Identification of target-binding peptide motifs

Based on MatLab built-in functions, we developed a script that groups peptides according to similarities. First, it calculates pair-wise distances among the peptides. It then constructs a phylogenetic tree using the distances calculated. Last, it clusters the peptides in suitable groups, with two optional parameters to fine tune this grouping (see Supplementary Data). This script allowed to efficiently identify target-specific binding motifs. The MatLab script generated well-arranged groups of around 3–20 peptides with high sequence similarity that can be analyzed and validated by eye.

**Figure 4.**
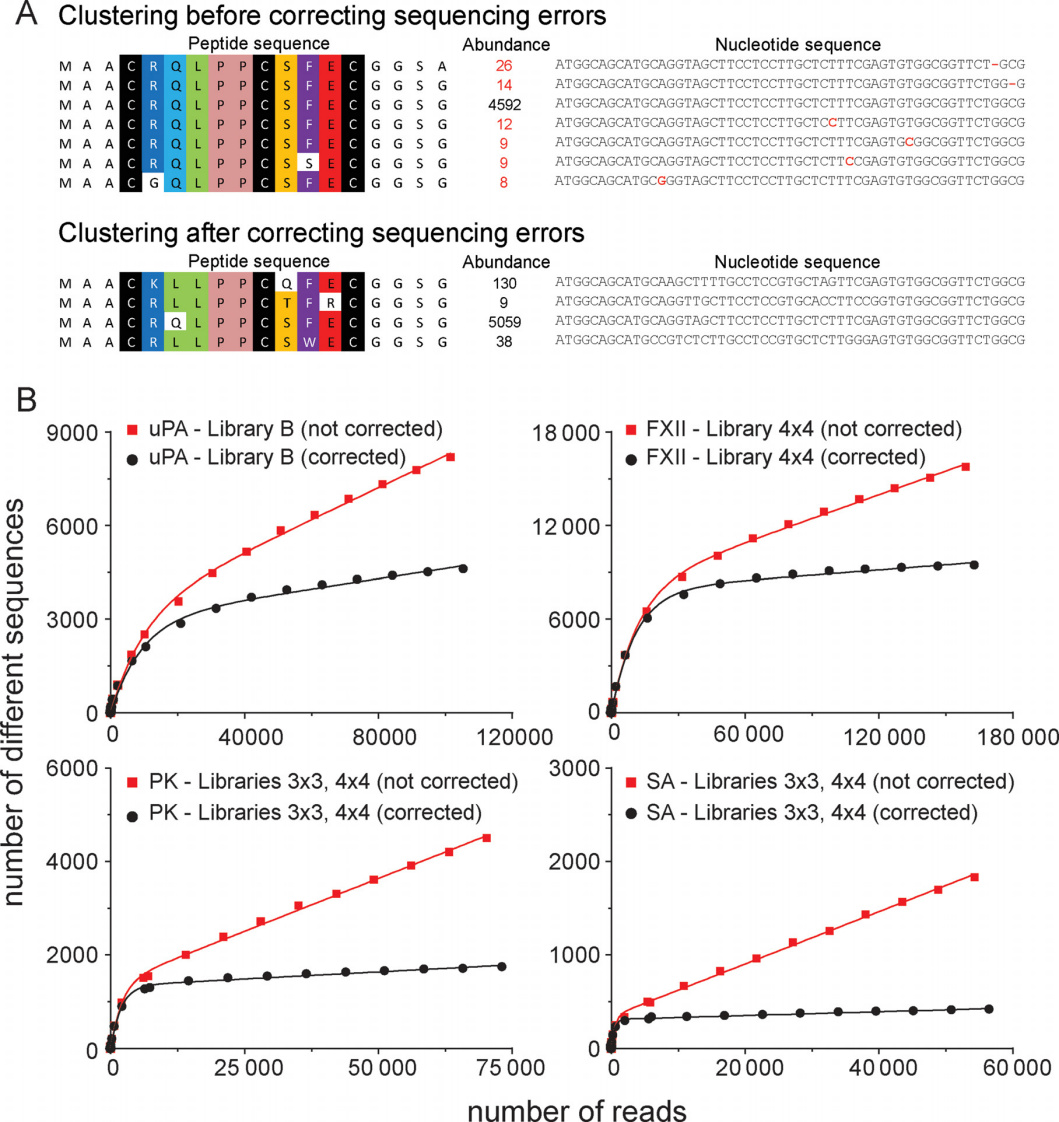
(**A**) Example for the identification of false consensus sequences due to sequencing errors. In a selection of Library A against SrtA, the most abundant sequence (present 4592 times) was clustered with sequences differing in only 1 nucleotide and being present at much lower frequency. These sequences likely resulted from sequencing errors. A MatLab script (*fixingerrors.m* script) was developed to eliminate these erroneous sequences. For the high-abundance sequence shown in the figure, 467 erroneous sequences were found (9%). For other high-abundance sequences, wrong sequences ranged between 0 and 48%. (**B**) Examples of saturation plots for different datasets before and after correcting sequencing errors (all datasets were obtained after one round of phage selection).

Inspection of consensus groups revealed that some of them were not true consensuses but artifacts that resulted from sequencing errors as explained in the following. For highly abundant peptides, peptide variants with nearly the same sequence were found. These peptides occurred in small copy numbers and typically differed in only one base from the abundant clone (e.g. insertion, deletion or mutation). An example from a selection against SrtA using library A is shown in Figure 4A: the most abundant clone was present 4592 times and several clones with similar DNA sequences appeared in only a few copies (ranging from 8 to 26). It is likely that the low-copy sequences resulted from sequencing errors because peptides with such small sequence differences are unlikely represented in the library. For example, library A contains only a small fraction (around 10^8^ different peptides) of the theoretically possible sequences (around 10^12^ sequence calculated from eight positions encoded by NNK codons). To eliminate sequencing errors and prevent false identification of consensus sequences, we developed a MatLab script that finds sequences that differ at only one or two positions and corrects them to the sequence of the more abundant clone. Indeed, application of this script led to elimination of a significant fraction of the errors. Consensus sequence artifacts were no longer found. Additionally, after this correction, parameter *b* in Equation (2) decreased below 1% (Figure 4B and Supplementary Table S2).

**Figure 5.**
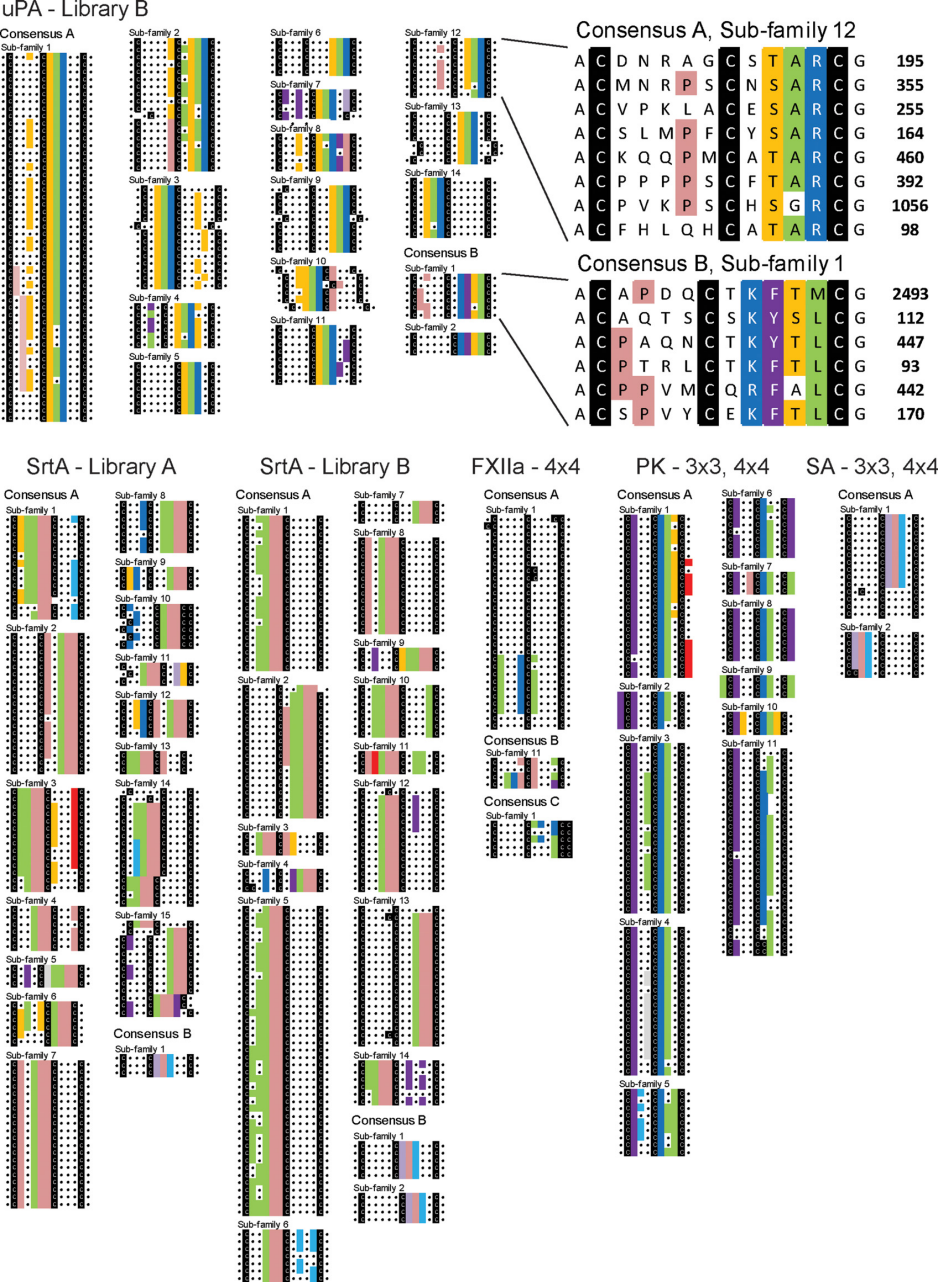
Identification of target-binding peptide motifs. The 200 most abundant peptides of each selection were computationally compared and clustered into groups of peptides that share a maximal sequence similarity. The raw data of the automated sequence comparison is included in the Supplementary Material. The sub-groups generated computationally were arranged manually to group those together that belong to the same consensus group. The cysteines are colored in black and regions in the peptides with sequence similarities were manually highlighted in color. Top: Consensus groups of peptides isolated in the selection with uPA. Peptide sequences of two of the sub-groups are enlarged and shown together with the abundance on the right side. Bottom: Consensus groups of peptides isolated against SrtA, FXIIa, PK and SA.

Consensus groups found after elimination of false sequences in the selections against all five protein targets are shown in Figure 5. As the required computational power increases quadratically with an increasing number of peptides, we compared only the top 200 abundant sequences from the different datasets. This was sufficient to identify consensus motifs in all selections. The analysis of larger numbers of sequences (up to 1000 sequences) did not lead to the identification of more target-binding motifs in this work (data not shown), but it may do if applied to other selections. In all phage selections performed, groups of peptides with high sequence similarities were found. Many of the groups formed by the MatLab script represented sub-families of a few entirely different consensus sequences. We manually highlighted the sequence similarities in all consensus groups with color (Figure 5).

Consensus sequences shared by only a small number of peptides were identified too. For example, the SA-binding motif HPQ was shared by as little as three different peptides in the SrtA selection and was still identified by the software. These peptides were isolated because biotinylated SrtA was immobilized on SA in the phage selection. In the uPA selection, the minor motif ‘^K^/_R_^F^/_Y_^S^/_T_L’ was shared by nine different peptides. The peptides could be assigned even to two different consensus sequence sub-families (Figure 5).

In all the selections, at least one or two target-binding peptide motifs could be found, namely ‘LPP’ for SrtA, ‘^T^/_S_AR’ and ‘^K^/_R_^F^/_Y_^S^/_T_L ’ for uPA, ‘VxxKCL’ for FXIIa, ‘^F^/_Y_\^W^ xxCRV’ for PK and ‘HPQ’ for SA (Table 2). The number of different consensus sub-families was much larger; it was 15 for SrtA, 16 for uPA, 2 for FXIIa, 11 for PK and 2 for SA. The motifs identified in selections with uPA, FXIIa, PK and SA were previously found by us or others after iterative rounds of phage selection and peptides with these motifs proved to be binders (19–21,25). In contrast, most of the consensus sub-families were previously not identified. The peptide motif ‘LPP’ found in selections against SrtA was not reported before; synthetic peptides with this motif bound to SrtA (results will be published elsewhere). Searching the whole pool of sequenced peptides for the identified target-binding peptide motifs revealed many additional sequences that are potential ligands of interest for characterization. Some consensus sequences contained up to around 2000 different peptides (e.g. in the uPA selection). Other contained as little as 93 different peptide sequences (FXIIa selection). In some selections, peptides with binding motifs represented more than 50% of the total number of sequenced peptides (uPA selection) or as little as 1% (FXIIa selection).

**Table 2.** Target-binding peptide motifs (patterns of conserved residues) found after one round of selection

Target	Library	Peptide motifs	Number of sub-families	Different peptides with motifs in top 200	Different peptides with motifs in whole pool	% of population containing a binding motif
SrtA	Library A	LPP	15	143 (72%)	1531 (41%)	47%
SrtA	Library B	LPP	14	164 (82%)	1253 (54%)	81%
uPA	Library B	^T^/_S_AR	14	165 (82%)	1943 (42%)	70%
		^K^/_R_^F^/_Y_^S^/_T_L	2	8 (4%)	44 (1%)	2.7%
FXIIa	4×4	RPCP	1	2 (1%)	23 (0.2%)	0.4%
		VXXKCL	1	5 (2%)	93 (1%)	1.2%
PK	3×3, 4×4	^F^/_Y_\^W^ XXCRV	11	90 (45%)	758 (43%)	43%
SA	3×3, 4×4	HPQ	2	17 (8.5%)	37 (9%)	7.4%

### Peptide motif identification from inter-dataset comparisons

In phage panning experiments, many phage particles are isolated unspecifically along with the peptides that are selectively isolated through binding to a target. If the number of specifically isolated peptides is small compared to the unspecific ones (named background phage), it is more difficult to identify specific target-binding sequences after just one round of selection. Additional rounds of phage selection may be needed. We hypothesized that, in such cases, a possible way to identify specific target-binding sequences in the presence of high background would be to perform two parallel selections and compare the sequences obtained. Identical peptides would be considered as target-specific peptides. We repeated a first round of selection against FXIIa and found that only six peptide sequences were common in both pools. Four of them corresponded to confirmed binding motifs that were previously found after three rounds of selection (Table 3) (21).

**Table 3.** Peptides identified by inter-dataset comparison

Abundance selection 1	Abundance selection 2	Peptide sequence	Peptides identified in previous phage selections^a^
307	12	ACDARPCPQTYCL	yes
40	110	QCVPLKCLWDRCE	yes
27	22	VCERQVCYLMSCW	no
12	36	TCLCKRCIKELCC	yes
11	16	YCVWDKCLWLMCE	no (but similar to consensus)
5	9	ACGMSICVLYGCN	no

^a^In previous phage selections, three iterative rounds of panning were performed and around 100 clones sequenced.

### Formats of isolated peptides

**Figure 6.**
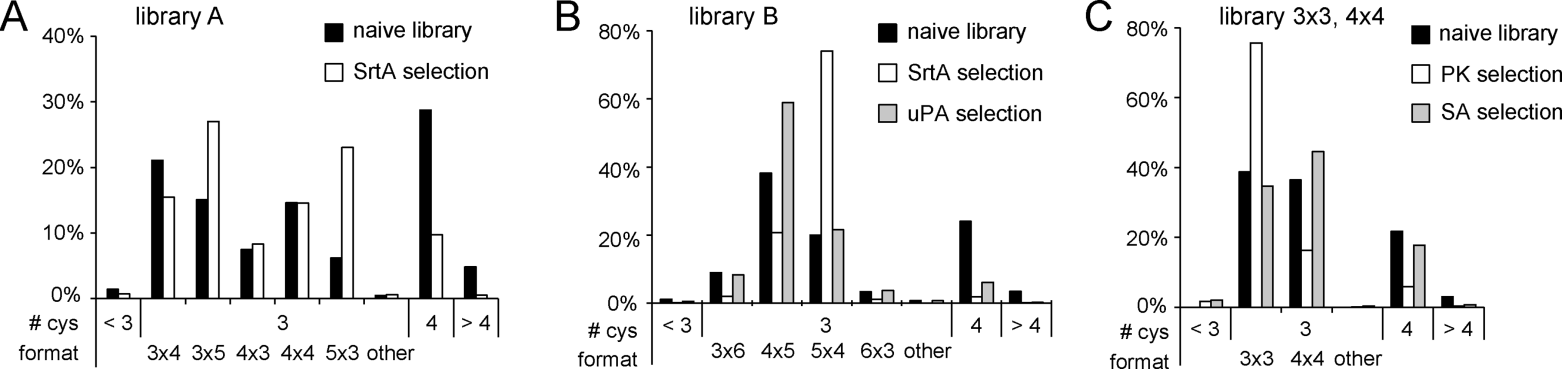
Statistical analysis of peptide formats using large sequence data. The percentage of peptides containing <3, 3, 4 or > 4 cysteine residues are indicated. For peptides containing three cysteines, the percentage of peptides with different formats are indicated. For example ‘3×4’ means that the peptides contain three amino acids between Cys1 and 2, and four amino acids between Cys2 and 3. (**A–C**) Results of selection with different targets and libraries. ‘Naive’ means the peptides in the library before selection.

The number of cysteines found in phage-selected peptides can indicate if they are forming linear, monocyclic or bicyclic peptide structures. We anticipated the isolation of peptides with three cysteines that are cyclized with TBMB and form bicyclic peptide structures. Occasionally, peptides with less or more than three cysteines are isolated from the applied phage peptide libraries. Previous work showed that peptides having a fourth cysteine residue in the randomized region are isolated as bicyclic peptides formed by two disulfide bridges (25). Due to errors in the library generation, some peptides have two cysteines and are isolated as disulfide-linked monocyclic peptides. Availability of the vast sequence data allowed detection of small differences in the number of cysteines and preferences for one or the other format in the different selections. In selections performed with libraries containing peptides of different ring sizes (number of amino acids spacing the cysteines), we analyzed if one or the other format was preferentially isolated. Peptides with certain ring sizes were preferentially enriched in selections with some protein targets. In the selection of SrtA binders from library A, bicyclic peptides of the formats 3×5 and 5×3 were enriched over other formats (Figure 6). Panning of library B against SrtA enriched bicyclic peptides of the format 5×4, while panning against uPA yielded more bicyclic peptides of the format 4×5 (Figure 6B). When the libraries 3×3 and 4×4 were mixed and panned against PK, 3×3 clones had a selective advantage, which was not the case when the same mix was panned against SA (Figure 6C).

### Iterative rounds of phage selection

**Figure 7.**
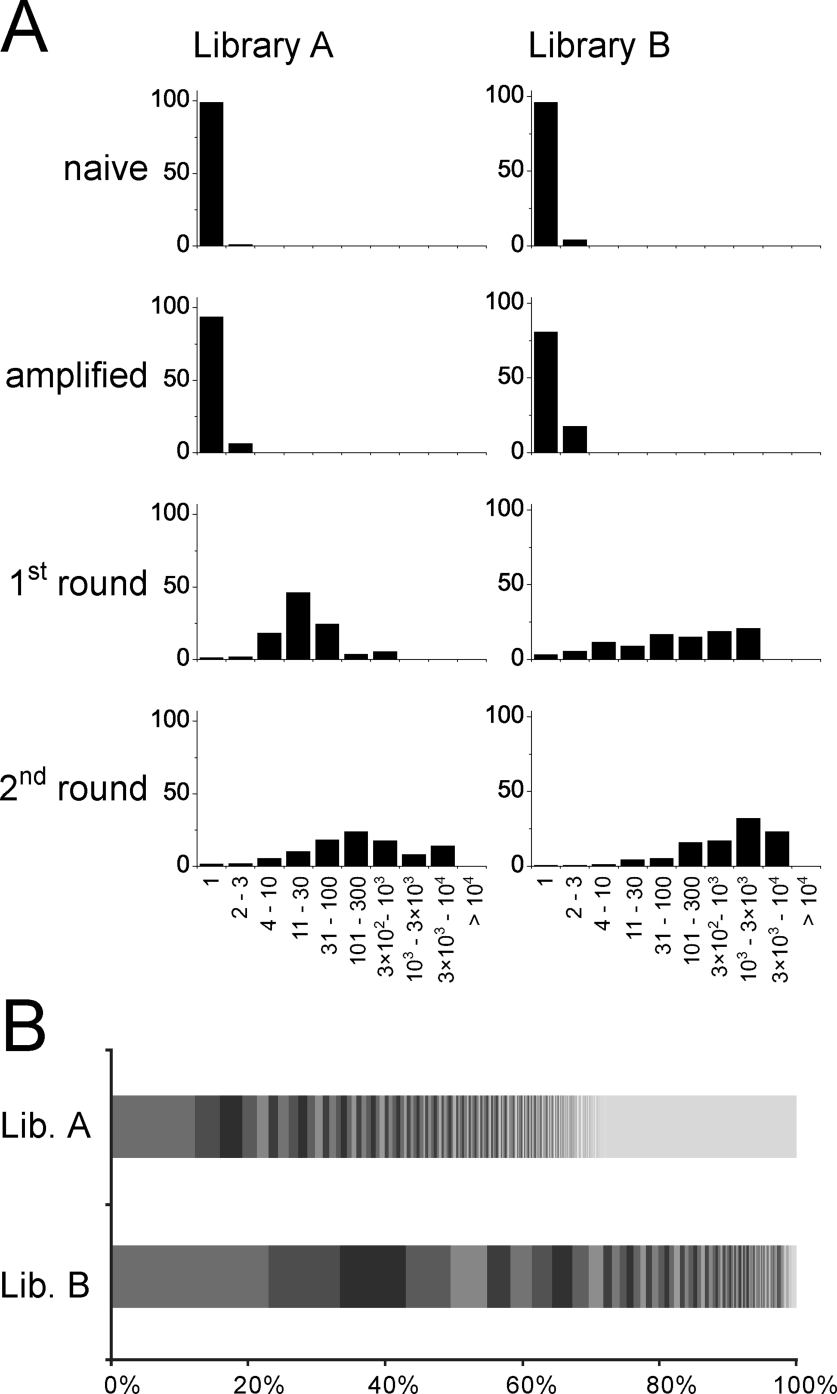
Dynamic change of the population over two rounds of selection. Results are shown for the selection of SrtA binders from libraries A and B. (**A**) Copy number of sequenced peptides. Indicated is the percentage of peptides that were identified at the indicated range. (**B**) Abundance distribution of the output of the second round. The most abundant 200 peptide sequences are separated in blocks.

We performed a second round of phage selection to study the population diversity (number of different sequences) and homogeneity (abundance distribution) over two rounds of selection. In particular, we were interested to learn (i) how many sequences with consensus motifs are lost in a second round of selection and (ii) if new sequences with binding motifs appear. Phage isolated from library A and library B against SrtA in the first round were subjected to a second round of affinity selection against SrtA and isolated clones sequenced. The population underwent a progressive loss of diversity over iterative rounds of selection (Figure 7). The number of different sequences decreased from 2800 (round 1) to 800 (round 2) in the case of library A, and from 1400 to 170 in the case of library B. In the selection with library A, around half (47%) of the peptides isolated in round 1 contained the ‘LPP’ motif and thus were binders. In round 2, nearly all the peptides (98.4%) were binders. Around one third of the sequences with the binding motif ‘LPP’ found in round 1 were lost in round 2. Interestingly, 22% of the population of the second round corresponded to sequences that were not found in the first round, indicating that the sampling of the first round was not complete and not all the diversity of the first round was sequenced. In the selection with library B, in the first round already 81% of the population of reads corresponded to binding sequences. After the second round, virtually all the population consisted in target-binding sequences (99.4%). A large fraction of the population after round 1 was also found in round 2 (71%), and new binding sequences found in the second round corresponded to less than 1% of the population.

## DISCUSSION

Sequencing of phage-selected peptides by high-throughput methods can offer a deep insight into the nature of selected peptides and the process of affinity panning and propagation. Pioneering studies in which phage-selected peptides were sequenced with high-throughput methods primarily used the data to study the peptide diversity and to identify highly abundant clones that are expected to bind with the highest affinities (10–16). Herein, we proposed to use HTS data to identify target-binding motifs as well as to obtain a more detailed picture of consensus sequences. A limitation we encountered was the lack of broadly applicable and flexible open-access computational tools to compare and analyze the sequences of a large number of peptides. We therefore devised a procedure and developed software that processes HTS data and that can identify consensus sequences.

In our strategy, phage-selected peptides are first ranked by their abundance and then compared pairwise to align peptides with sequence similarities. The software reads sequence raw data from fastq files that are provided by most HTS platforms. The output of the tool are groups of 3–20 peptides sharing sequence similarities. Importantly, the software is keeping the information about the abundance and nucleotide sequence of each peptide sequence and displays this information in the analysis result. The software can deal with commercially available as well as self-tailored libraries. It includes functionalities for analysis of specific library formats such as disulfide-cyclized peptides or bicyclic peptides. Additional functions allow inter-dataset comparisons as well as searching for peptides containing specific sequence motifs.

While developing the analysis procedure and software, we learned that it is important to understand biases introduced by next-generation sequencing technologies. It is paramount to optimize quality filters to prevent introduction of biases. The main error source in Ion Torrent PGM sequencing is inaccurate flow-calls, which result in insertion/deletion (indel) errors, most frequently in homopolymeric regions (28,29). Even correctly called homopolymeric regions are typically assigned less confidence (27). Filters applied inappropriately could remove too many sequences and in this way introduce strong biases. We empirically identified an optimal quality filter which tolerates three bases with qualities below Q18. This filter gave the best result for all selections presented in this work and most likely is suitable for analysis of peptides isolated from any other type of combinatorial peptide library.

Sequencing errors were found to mislead standard algorithms that are used to identify sequence similarities and consensus sequences. We show that sequencing errors on highly abundant clones produce a series of erroneous variants, whose abundance is generally lower. The abundant clone together with a group of similar erroneous sequences were recognized by the software as a consensus group. We developed a procedure that eliminates sequencing errors from the dataset. False sequences are identified as such if they have identical nucleotide sequences except for one or two positions. Application of this filtering procedure eliminated the identification of false consensus sequences.

Our software was able to identify consensus sequences and sub-families of consensus sequences in datasets of all phage selections. Even consensus motifs that were shared by only a few peptides in the population could be identified. As we compared only the most abundant 200 peptides in each selection, some consensus motifs were most likely missed. More target-binding motifs may be identified if significantly more peptides are compared. The scripts were run on a standard personal computer within minutes. Thousands of sequences may be compared by using high performance computers. In the analyzed 200 sequences per selection, 1–3 consensus sequences were found that were further divided into many sub-families with slight consensus variations. This finding indicated that most proteins have only one or at most few regions where peptides can bind with sufficiently high affinity allowing their isolation. This is in contrast to antibodies that typically bind to more different epitopes.

An important parameter in the phage selection is the copy number of the peptides that are subjected to affinity selections. Only if a peptide is available in the library in a sufficiently large copy number, it can be isolated and sequenced in multiple copies and appears as an ‘enriched’ peptide. In some of the selections performed in this work, the average copy number of the peptides was rather low and the isolated peptides diverse. The identification of target-binding peptide motifs was thus difficult. For example in the selections against PK and SA, the average copy number was 2. Consensus sequences could in these cases only be identified because many peptides were sharing the same motif.

In selections with more challenging targets such as FXIIa, it was difficult to identify target-binding motifs. Only 1% of all peptides isolated against FXIIa contained FXIIa-binding motifs (some of the motifs were known from previous work). Most of the 99% remaining sequences are most likely peptides that were isolated through non-specific interactions. Our software could nevertheless identify two consensus sequences. We also investigated the possibility of reliably identifying specific-binding sequences by performing in parallel independent selections. We reasoned that this approach could allow the identification of specific target-binding ligands from noisy datasets and for the identification of parasitic sequences. By comparing the output of two selections performed in parallel against FXIIa (one selection round), we indeed could differentiate specific target-binding clusters from background clusters. Inter-dataset comparison may also be applied to identify peptides that bind to the SA magnetic beads rather than to the protein target.

Our work confirmed that peptide ligands can be efficiently identified in a single round of phage selection if isolated clones are analyzed by HTS. In contrast to previous work that identified peptide ligands based on their abundance, we show that extensive comparison of sequences can identify additional attractive ligand candidates. Phage selection of peptide ligands in a single instead of multiple rounds has also the advantage that propagation-related bias is reduced to a minimum (11,30,31). This could be particularly important when genetically engineered phage systems containing unnatural amino acids are used (32). Finally, a single round of phage panning may also facilitate the application of phage display by scientist that have no prior experience with this technique. Readily prepared libraries could simply be pipetted to a target and captured phage sequenced. Phage amplification and purification would not be necessary, and equipment for bacteria culture and phage handling would not be required.

In summary, we have developed a strategy and software to compare large numbers of phage-selected peptides that were sequenced by high-throughput methods. With this strategy, we were able to identify rare target-binding peptide motifs, as well as to define more precisely consensus sequences and sub-groups of consensus sequences. This information is valuable to choose peptide leads for drug development and it facilitates identification of epitopes.

## SUPPLEMENTARY DATA

Supplementary Data are available at NAR Online.

SUPPLEMENTARY DATA
