# Supplementary material for: Identification of target-binding peptide motifs by high-throughput sequencing of phage-selected peptides
Source: Nucleic Acids Res. 2014 Oct 27;42(22):e169. doi: 10.1093/nar/gku940 (PMC4267670; doi:10.1093/nar/gku940)
Supplement: SUPPLEMENTARY DATA [file supp_42_22_e169__index.html]

Identification of target-binding peptide motifs by high-throughput sequencing of phage-selected peptides — SUPPLEMENTARY DATA 

# Identification of target-binding peptide motifs by high-throughput sequencing of phage-selected peptides

## SUPPLEMENTARY DATA

**Files in this Data Supplement:**

- SUPPLEMENTARY DATA
- SUPPLEMENTARY DATA
