# Supplementary material for: Identification of target-binding peptide motifs by high-throughput sequencing of phage-selected peptides
Source: Nucleic Acids Res. 2014 Oct 27;42(22):e169. doi: 10.1093/nar/gku940 (PMC4267670; doi:10.1093/nar/gku940)
Supplement: SUPPLEMENTARY DATA [file supp_gku940_Supplementary_Data_NAR-01378-Met-K-2014_revised.pdf]

Supplementary Data

Identification of target-binding peptide motifs by high-throughput sequencing of phage-selected peptides

Inmaculada Rentero Rebollo*, Michal Sabisz, Vanessa Baeriswyl and Christian Heinis*

Supplementary results

Barcode assignment pg. 2

Validation of the mathematical model to estimate the number of different sequences pg. 3

Supplementary tables

Supplementary Table S1 pg. 4

Supplementary Table S2 pg. 5

Supplementary figures

Supplementary Figure S1 pg. 6

Supplementary Figure S2 pg. 7

Supplementary Figure S3 pg. 8

Supplementary Figure S4 pg. 9

Supplementary Figure S5 pg. 10

MatLab script description

Step1 pg. 11

Step2 pg. 12

LoopLengths pg. 14

Clustering pg. 15

FindSeq pg. 16

CommonSeq pg. 17

Supplementary results

Barcode assignment

Barcodes were designed so they could be identified even if one or two of the six bases were wrongly sequenced. We included in the MatLab script an option to allow one mismatch in the barcode (one insertion, mutation or deletion). Application of this procedure did not increase much the number of sequences that could be used because most of the rescued sequences were filtered out in the subsequent quality filter due to bad quality values in the peptide region (Supplementary Figure S1). For all the analysis in this work, we therefore used only sequences in which the barcode showed a perfect match.

Validation of the mathematical model to estimate the number of different sequences

For a homogeneous population of sequences (where all clones are equally represented in the pool), the number of different sequences found (*y*) in function of the number of sequences sampled (*x*) will increase linearly at the beginning, decreasing the rate as it approaches saturation (Supplementary Figure S2). This system could be approximated by equation 1

$$(a - y) = \mathrm{k}\frac{dy}{dx} \quad (1)$$

where *a* is the total number of different sequences in the pool, and the rate of finding new sequences (dy/dx) is proportional to the number of new sequences remaining in the sample, with a proportionality constant *k*. Solving the differential equation, the number of different sequences corresponds to equation 2:

$$y = a\left(1 - e^{-x/k}\right) \qquad (2)$$

Taylor approximation near 0, where the function behaves almost linearly (x << a), allows the determination of the initial slope of the curve as shown in equation 3:

$$y = \frac{a}{k}x,\ for\ x \ll a \qquad (3)$$

In the case of a homogeneous dataset, the initial slope should be close to one, and therefore $a = k$. Indeed, simulation of an ideal homogeneously distributed dataset gave the expected curve and fitted parameters corresponded to the ones simulated (Supplementary Figure S2).

In the case of non-homogeneous datasets, where a few sequences might represent a significant fraction of the population, we anticipated that the system would behave similarly, but with a lower initial slope. We validated this approach by fitting a series of simulated datasets representing populations with different abundance distributions (Supplementary Figure S3). The parameter a/k takes values between 0 and 1, and could be used to quantify the homogeneity of the sample:

(less homogeneous) $0 < a/k \leq 1$ (homogeneous)

In the experimental datasets, at larger number of reads, the number of different sequences increased linearly and did not converge to a maximal value. The linear increase was due to sequencing errors, which were directly proportional to the number of reads. To give account for this effect, a linear component was added to equation 2:

$$y = a\left(1 - e^{-x/k}\right) + bx \quad (4)$$

where *b* is a global error rate for the population. Equation 4 was used to fit the data in this study. We additionally simulated the same sets presented in Supplementary Figure S3, adding different percentages of random mutations, and using equation 4 to fit the data, obtaining a good estimation of the parameters (Supplementary Figure S4).

Supplementary Tables

Primer name	Sequence
Primers for PCR amplification of Library A and Library B	
IT_Fw1	5' CCATCTCATCCCTGCGTGTCTCCGACTCAGGCATAGTTTCTATGCGGCCCAGC 3'
IT_Fw2	5' CCATCTCATCCCTGCGTGTCTCCGACTCAGCGTATCTTTCTATGCGGCCCAGC 3'
IT_Fw3	5' CCATCTCATCCCTGCGTGTCTCCGACTCAGATCGCATTTCTATGCGGCCCAGC 3'
IT_Fw4	5' CCATCTCATCCCTGCGTGTCTCCGACTCAGACGATATTTCTATGCGGCCCAGC 3'
IT_Fw5	5' CCATCTCATCCCTGCGTGTCTCCGACTCAGAGACTCTTTCTATGCGGCCCAGC 3'
IT_Fw6	5' CCATCTCATCCCTGCGTGTCTCCGACTCAGGATACATTTCTATGCGGCCCAGC 3'
IT_Fw7	5' CCATCTCATCCCTGCGTGTCTCCGACTCAGCATCTCTTTCTATGCGGCCCAGC 3'
IT_Fw8	5' CCATCTCATCCCTGCGTGTCTCCGACTCAGGTTCAGTTTCTATGCGGCCCAGC 3'
IT_Fw9	5' CCATCTCATCCCTGCGTGTCTCCGACTCAGTACCAGTTTCTATGCGGCCCAGC 3'
IT_Fw10	5' CCATCTCATCCCTGCGTGTCTCCGACTCAGATGGAGTTTCTATGCGGCCCAGC 3'
IT_Rev1	5' CCTCTCTATGGGCAGTCGGTGATGTTTCAGCGCCAGAACC 3'
Primers for PCR amplification of Library 3x3 and Library 4x4	
IT_Fw11	5' CCATCTCATCCCTGCGTGTCTCCGACTCAGAGTTACCGCAATTCCTTTAGTTGTTC 3'
IT_Fw12	5' CCATCTCATCCCTGCGTGTCTCCGACTCAGGGTGAACGCAATTCCTTTAGTTGTTC 3'
IT_Rev2	5' CCTCTCTATGGGCAGTCGGTGATTTTCAACAGTTTCAGCGGAGTG 3'

Table S1. Primers for PCR amplification and subsequent Ion Torrent sequencing. Forward primers contain adaptor sequence, barcode (underlined) and template-specific sequence. Reverse primers contain adaptor sequence and template-specific sequence.

dataset	before correcting sequencing errors			after correcting sequencing errors		
	**a** (# different sequences)	**a/k** (homogeneity)	**b** (error rate)	**a** (# different sequences)	**a/k** (homogeneity)	**b** (error rate)
SrtA – Library A	2817	0.743	3.3%	2814	0.712	1.8%
SrtA – Library B	1442	0.212	2.8%	1422	0.222	1.4%
uPA – Library B	3129	0.319	5.1%	2980	0.321	1.6%
FXII – 4x4	7884	0.671	5.1%	7839	0.719	1.1%
PK – 3x3, 4x4	1376	0.670	4.5%	1333	0.704	0.6%
SA – 3x3, 4x4	343	0.641	2.8%	311	0.725	0.2%

Table S2. Correcting errors in sequences of phage-selected peptides. Diversity, homogeneity and error rate were estimated before and after correcting sequencing errors for different datasets (datasets were obtained after one round of phage selection and the correction was applied to all sequences). The estimated error rate (parameter *b*) is considerably reduced, while the estimated number of different sequences and homogeneity remain almost unchanged.

Supplementary Figures

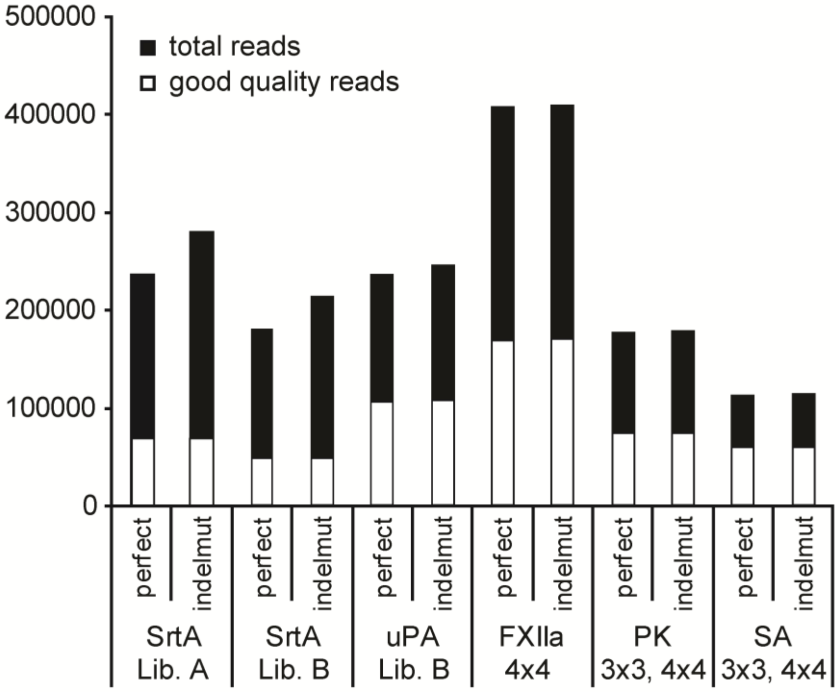

Figure S1. Number of sequences found for each barcode. Bars labeled with 'perfect' show the number of sequences having a perfect match of the barcode. Bars labeled with 'indelmut' show the sum of sequences with a perfect match and those having one insertion, deletion or mutation in the barcode. The white area within the bars shows sequences that passed the quality filter after analyzing the peptide region (quality parameters: maximum 3 bases < Q18).

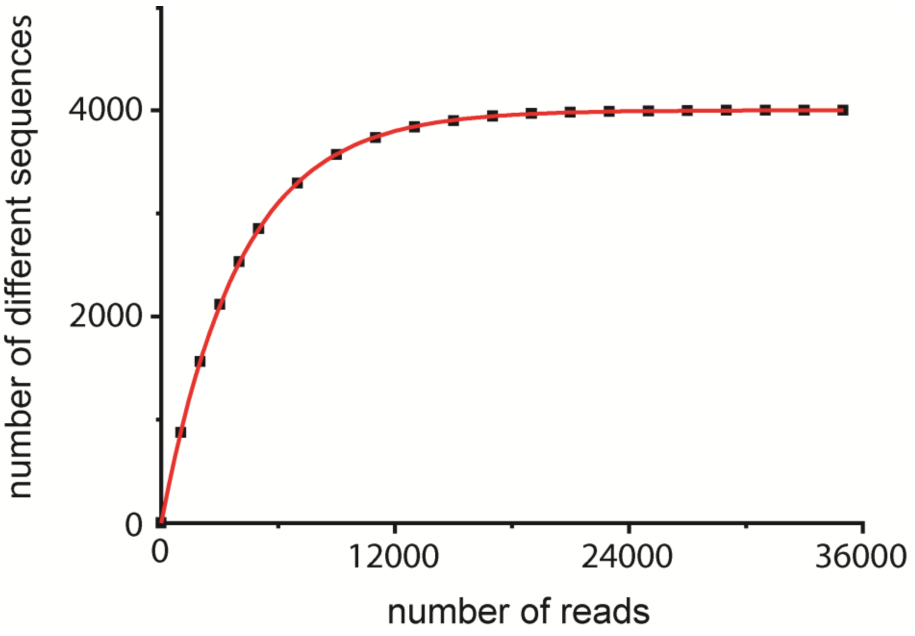

Figure S2. Simulation of an ideal dataset of homogeneously distributed sequences. The dataset was chosen to contain 4000 different sequences. The number of different sequences are indicated in dependence of the number of reads sampled (black squares). Equation 2 was used to fit the data (red line). Calculated parameters (total number of different sequences = 4000, *a/k* = 0.995) corresponded with the ones simulated.

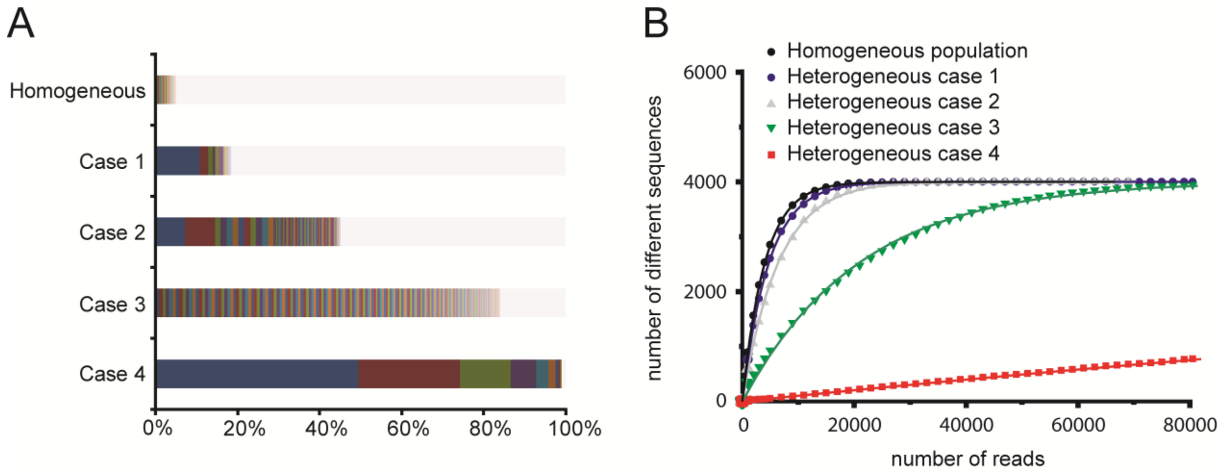

C

dataset	a (total different sequences)	a/k (homogeneity)
Homogeneous	4000	0.995
Case 1	4001	0.843
Case 2	3995	0.572
Case 3	4012	0.191
Case 4	4137	0.01

Figure S3. Simulation of datasets presenting different abundance distributions of peptide sequences. (**A**) Representation of the homogeneous population and different simulated heterogeneous populations (case 1 to case 4). All contained a total of 4000 different sequences but they were present in different relative abundances. Top 200 most abundant sequences are separated as blocks shown in different colors. (**B**) Saturation plots of these heterogeneous populations and subsequent fitting of equation 2. (**C**) Calculated parameters from the fitting. For case 4, 10^6 reads were sampled to reach saturation.

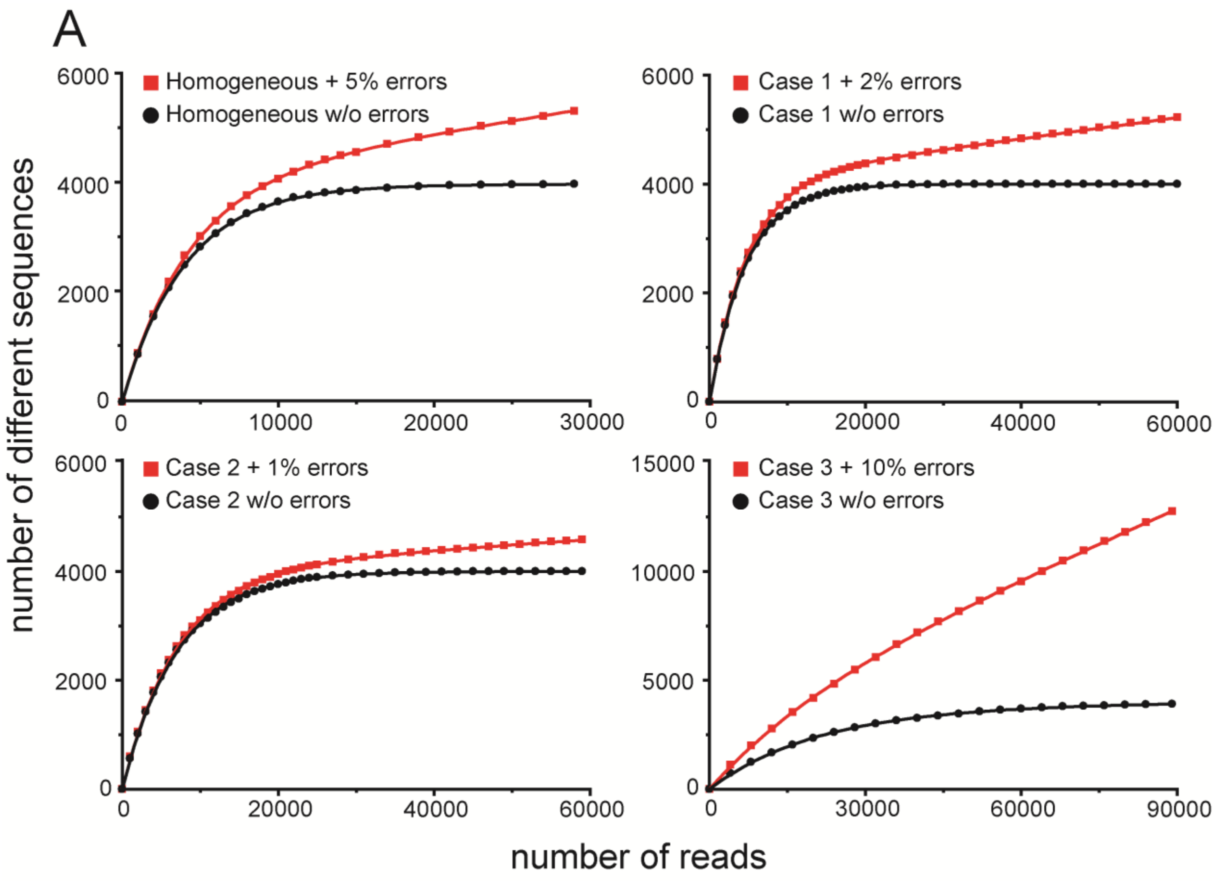

B

dataset	a (total different sequences)	a/k (homogeneity)	b (error rate)
Homogeneous + 5% errors	4005	0.961	0.0464
Case 1 + 2% errors	4052	0.854	0.0195
Case 2 + 1% errors	3995	0.572	0.0098
Case 3 + 10% errors	3411	0.165	0.106

Figure S4. Simulation of populations containing 4000 different sequences and presenting different abundance distributions and different sequencing error rates. (**A**) Saturation plots of simulated populations and fitting of equation 4. (**B**) Calculated parameters from the fitting. Good estimates for the total number of different sequences *a*, the error rate *b* and the homogeneity of the population a/k were obtained.

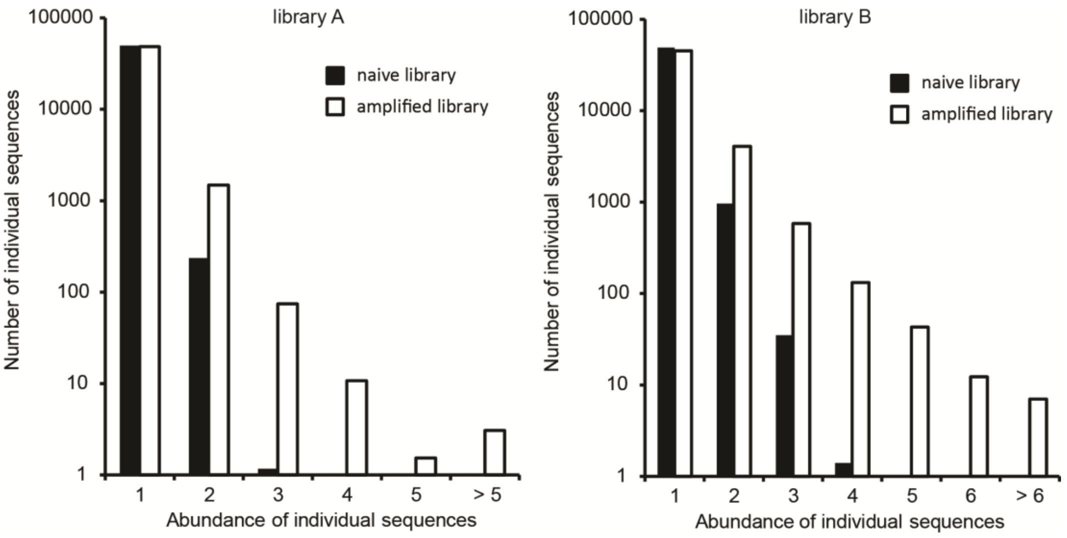

Figure S5. Libraries analyzed before (naive library) and after one round of amplification (infection of bacteria without affinity selection; amplified library). A sub-population of the library is being preferentially amplified, but the most abundant clone represents less than 0.02% of the population.

MatLab Scripts description

A step by step manual is provided together with the MatLab scripts in a compressed folder.

Step1.m

- It reads the initial filename.fastq file and generates files containing reads according to their barcode (named BC1.txt, BC2.txt... BCNOT.txt), and saves them in a separate folder within the input folder (named "filename_BC"). BCNOT.txt contains reads whose barcode did not correspond to any of the identified barcodes. If no input is indicated, a dialog box opens to choose the file and the barcodes used are the 12 described in this publication.
- Input: (optional)
    - Step1('inname','filename.fastq','indir','path') indicates the file name and path to the folder where it is located. If not specified, a dialog box to choose the file will open.
    - Step1(...,'indelmut','on') allows one insertion, deletion or mutation in the barcodes. If not specified, it is off.
    - Step1(...,'bc',{'AAAAAA',' TTTTTT','GGGGGG';'...'}) indicates the barcodes used. They must be separated by comma, in single bracket and within {}. If not specified, it uses the ones described in this publication (Table S1).
- Output:
    - Command window:
        - chip-specific code
        - time taken to read different fractions of filename.fastq
    - A new folder called "filename_BC" with a series of files named BC1.txt, BC2.txt... BCn.txt, containing the reads corresponding to the first, second... nth barcode respectively. Reads whose barcode did not correspond to any of the identified barcodes are stored in BCNOT.txt.
    - BC_stats.txt file, containing information about how many reads where found per barcode.

Step2.m

- It removes low quality reads from the datasets, groups identical DNA sequences and sorts them by abundance. It then translates them (amber codon is translated to glutamine). If no input is specified, a dialog box opens that allows choosing the file (a BCn.txt output of Step1). The constant DNA sequences flanking the random region must be indicated (if not indicated, start and end of the random region are the ones suitable for bicyclic peptide libraries used in this publication). Default quality parameters are 3 base calls below Q18. Optionally, a minimum, intermediate and maximum length of the peptide can be indicated. The additional correcting error step is recommended for low-diversity datasets in which a few clones predominate in the library, but it may take 10-20 minutes. It will merge together sequencing having one or two different positions in the DNA sequence.
- input (optional):
  - Step2('inname','filename.txt','indir','path') indicates the file name and path to the folder where it is located. If not specified, a dialog box to choose the file will open. Step2 can read the output file from Step1 (BC1.txt, BC2.txt...).
  - Step2(...,'badmax',n) where n is the maximum number of bases below the quality threshold allowed. If not specified, badmax = 3
  - Step2(...,'q',Q) where Q indicates the quality threshold (18 for Q18, 20 for Q20, etc...). If not specified, Q = 18.
  - Step2(...,'uplimit',m,'downlimit',o,'midlimit',p) specifies the maximum (m) and minimum (o) peptide length (in residues). Additionally, an internal limit can be indicated (p).
  - Step2(...,'start','NNKNNK','end','NNKNNK') specifies constant regions at the start and end of the DNA region of interest. If not specified, it uses the ones described in this publication. The first nucleotide of 'start' must be the first nucleotide of the codon for the translation to be in frame.
  - Step2(...,'fixerr',n) allows to correct sequencing errors: it merges together sequencing with only 1 or 2 differences in the DNA sequence. It corrects only the top "n" abundant sequences.
- output:
  - Translation_filename folder containing the file Translated_filename_GOOD.txt and Translated_stats.txt (indicating the number of different sequences, maximum abundance and total number of reads).
  - Optionally: additional files folder within the previous folder containing the translation files of the bad quality reads, or too-long or too-short reads.
  - Optionally: QF_filename folder with QF_filename_GOOD.txt and QF_filename_BAD.txt containing good and bad quality reads respectively. QF_filename_NOLIM.txt contains reads where either the start or the end of the region of interest could not be found. QF_filename_toolong.txt and QF_filename_tooshort.txt contain the reads whose peptides where shorter or longer than the limits indicated. If

an intermediate limit was indicated, two files: a QF_filename_longGOOD.txt and a QF_filename_shortGOOD.txt, are created. IDEM with BAD.

- If 'fixerr' option is on, within the Translation_filename folder, a file called "fixerrTranslated_filename.txt" appears, as well as an additional folder "correction data". In this folder there are files with all the correction events (potential conflict ones are in a separate file for an easier evaluation, i.e. if the abundances differ in less than 4x). ErrorRates.txt contains the error rate (1st column), the starting occurrence (2nd column) and the final occurrence after correcting (3rd column) of the peptides.

LoopLengths.m (for monocyclic and bicyclic peptide libraries)

- Separates sequences in different files according to the peptide format (i.e. number of cysteines and the number of residues between them). If no input is specified, a dialog box opens that allows choosing the file (having the format: peptide seq - abundance - nucleotide seq). Optionally, one can indicate a minimum abundance for a sequence to be considered, and a constant C-terminal peptide sequence to remove frame-shifted sequences.
- Input (optional):
  - LoopLengths('inname','filename.txt','indir','path') indicates the file name and path to the folder where it is located. If not specified, a dialog box to choose the file will open. LoopLengths needs a file with data on the format: peptide seq - abundance - nucleotide seq.
  - LoopLengths(...,'cutoff',n), where n specifies the minimum abundance to be considered. If not specified, no cutoff is applied and all sequences are considered.
  - LoopLengths(...,'cter','XXX'), where XXX is the amino acid sequence found at the C-terminus of the peptide. Allows to remove frame-shifted clones.
- Output:
  - A new folder called "LoopLengths_filename" with 4 subfolders:
    - 2cys: containing files with the sequences corresponding to 2 cysteines, subdivided by loop lenght. Example of the notation:
      3_twocys = C XXX C
      5_twocys = C XXXXX C
    - 3cys: containing files with the sequences corresponding to 3 cysteines, subdivided by loop lenght. Example of the notation:
      3_threecys = 0x3 = CC XXX C
      300_threecys = 3x0 = C XXX CC
      304_threecys = 3x4 = C XXX C XXXX C
      305_threecys = 3x5 = C XXX C XXXXX C
    - 4cys: containing files with the sequences corresponding to 4 cysteines, subdivided by loop lenght. Example of the notation:
      4_fourcys = 0x0x4 = CCC XXXX C
      400_fourcys = 0x4x0 = CC XXXX CC
      403_fourcys = 0x4x3 = CC XXXX C XXX C
      40000_fourcys = 4x0x0 = C XXXX CCC
      40302_fourcys = 4x3x2 = C XXXX C XXX C XX C
    - other: containing files with the sequences corresponding to 0, 1 or more than 4 cysteines; and "stats" file with the information about the number of total and different sequences assigned to each category.

Clustering.m

- Compares a chosen number of sequences (if not specified, compares top 200) and groups them into families that share high sequence similarity. Within a cluster, more similar sequences appear together. A figure logo for each group is generated and saved as a .jpg file within the input folder. Optionally, the number of different sequences OR the minimum abundance can be indicated. If no input is indicated, a dialog box will open that allows choosing the file. It must be a file of the format: format peptide seq. – abundance - nucleotide seq. Additionally, two optional parameters allow fine-tuning of the clustering: "min_clustersize" and "stringency".
- Input (optional):
  - Clustering('inname','filename.txt','indir','path') indicates the file name and path to the folder where it is located. If not specified, a dialog box to choose the file will open. Clustering can read the output files from Step2, LoopLengths and FindSeq. Requisites: data is on the format peptide seq – abundance - nucleotide seq.
  - Clustering(...,'number_dif',n) indicates how many different sequences will be clustered. If not specified, n = 200, i.e. top 200 most abundant sequences will be clustered.
  - Clustering(...,'min_clustersize',m) indicates the minimum number of sequences within a cluster to be considered. If a cluster has less than m sequences, it will be transferred to the "mixed" cluster.
  - Clustering(...'cter','XXXX') indicates a constant C-terminal region of the peptide. Peptides without this constant region will be not considered.
  - Clustering(...,'min_abun',n) indicates the minimum abundance for clones to be considered.
  - Clustering(...,'stringency',s) allows to fine-tune the clustering of the script to different datasets. In general, higher values of stringency will lead to more similar peptides within each cluster and more sequences in the mixed cluster. Lower values of stringency allow more differences within each cluster and as a result fewer sequences go to the mixed cluster.
  - Clustering(...,'logos','off') disables the generation of sequence logos (.jpg files) within the input folder.
  - Clustering(...'gappen',n) changes the value of gap opening and gap extension penalties. Default value is 8.
- Output:
  - Clusters_filename.txt file within the same folder as the input file.
  - A series of .jpg files corresponding to the sequence logos of each group within the same folder as the input file.

FindSeq.m

- Searches the dataset for all peptide sequences containing a specified motif. The motif must be specified in the input, and can be a string of characters or a regular expression. It distributed the peptides in two different files, according to whether they contain the specified motif or not.
- Input:
  - FindSeq('seq','XXX'), will look for XXX motif. For example, FindSeq('seq','HPQ') will look for all sequences in the dataset containing HPQ. Regular expressions can be used instead, for example, FindSeq('seq','H.Q') will look for all sequences conatining HXQ, X being any amino acid. FindSeq('seq','H.?Q') will look for all sequences containing HQ or HXQ, X being any amino acid. For more informaiton about regular expressions, see MatLab help.
- Input (optional):
  - FindSeq('inname','filename.txt','indir','path') indicates the file name and path to the folder where it is located. If not specified, a dialog box to choose the file will open. Requisites: data is on the format peptide seq – abundance - nucleotide seq.
  - FindSeq(...'cter','XXXX') indicates a constant C-terminal region of the peptide. Peptides without this constant region will be not considered.
  - FindSeq(...,'cutoff',n) indicates the minimum abundance for clones to be considered.
- Output:
  - A new folder named "Seq" within the input folder, containing three files: Seq_XXX_match.txt (XXX is the specified regular expression where especial characters have been substituted by "_"), containing all sequences that match the expression. Seq_XXX_nomatch.txt, containing the sequences that do not match it. And Seq_XXX_stats.txt, containing how many total and different sequences were assigned to each file.

CommonSeq.m

- Compares up to three different datasets and distributes common and exclusive sequences in different files.
- Input (optional):
  - CommonSeq('inname1','filename1.txt','inname2','filename2.txt','inname3','filename3.txt','indir1,'path1','indir2','path2','indir3','path3') specifies three files and three paths corresponding to them. If not specified, dialog boxes will open for each.
  - CommonSeq(...,'cutoff',n), where n is the minimum abundance to be considered
  - CommonSeq(...,'top',m), alternative to the previous one, it indicates the top m abundant sequences of each file will be considered
  - CommonSeq(...,'cter','XXX'), specifies constant C-terminal residues (allows the removal of frame-shifted clones that do not have them)
- Output
  - A new folder named "comparison" within the folder containing the FIRST FILE. The following files are generated:
    - Comparison_seq1.txt, Comparison_seq2.txt, Comparison_seq3.txt = contain sequences that appeared only in the first, second and third file respectively
    - Comparison_seq12.txt, Comparison_seq13.txt, Comparison_seq23.txt = contain sequences that appeared in two of the files
    - Comparison_seq123.txt = contains sequences that appeared in the three files
    - Comparison_stats.txt = contains the number of total/different sequences considered in each case and the number of different sequences assigned to each file
