# Supplementary material for: Identification of target-binding peptide motifs by high-throughput sequencing of phage-selected peptides
Source: Nucleic Acids Res. 2014 Oct 27;42(22):e169. doi: 10.1093/nar/gku940 (PMC4267670; doi:10.1093/nar/gku940)
Supplement: SUPPLEMENTARY DATA [file supp_gku940_nar-01378-met-k-2014-File009.zip › MatLab Scripts/ScriptsManual.pdf]

MANUAL

Identification of target-binding peptide motifs by high-throughput sequencing of phage-selected peptides

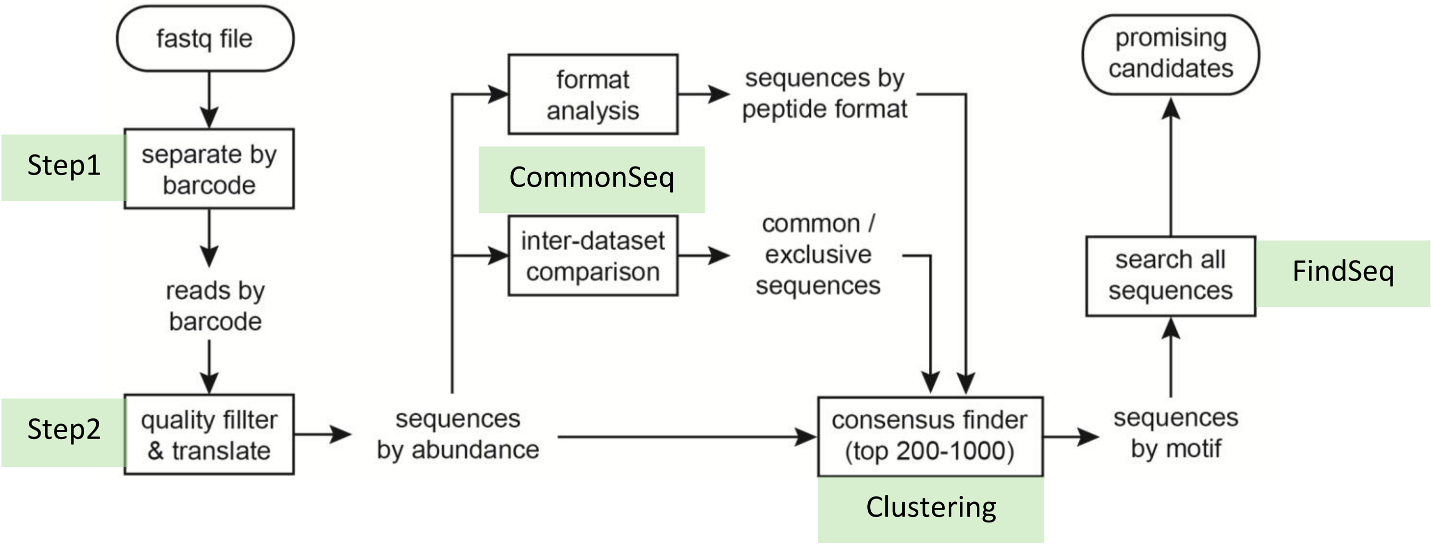

1. Before starting ____ *3*

How to work with MatLab (just the basics you need to know): ____ 3

2. Step1: separating files by barcodes ____ *4*

Function information ____ **4**

Running the function ____ **4**

Output ____ **5**

Advanced options ____ **5**

3. Step2: quality filtering and translation ____ *7*

Function information ____ **7**

Running the function ____ **7**

Output ____ **7**

Advanced options ____ **8**

4. Clustering ____ *12*

Function information ____ **12**

Running the function ____ **12**

Output ____ **13**

Advanced options 14

5. LoopLengths (for cys-constrained monocyclic or bicyclic peptides) *15*

Function information 15

Running the function 15

Output 15

Advanced options 16

6. CommonSeq *17*

Function information 17

Running the function 17

Output 17

Advanced options 18

7. FindSeq *19*

Function information 19

Running the function 19

Output 19

Advanced options 19

1. Before starting

- Install MatLab in your computer

- Create a folder exclusively for Ion Torrent

- Copy the folder "MatLab_Scripts" in the previous folder

For each dataset:

- Create a folder within the Ion Torrent for the present run

- Copy the sequence .fastq file in the previous folder

How to work with MatLab (just the basics you need to know):

When you open MatLab it will look like this:

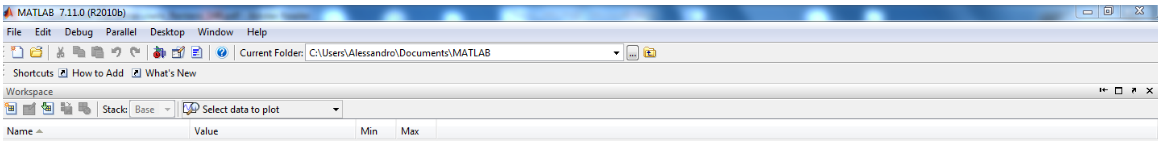

Workspace window not needed: you can close it

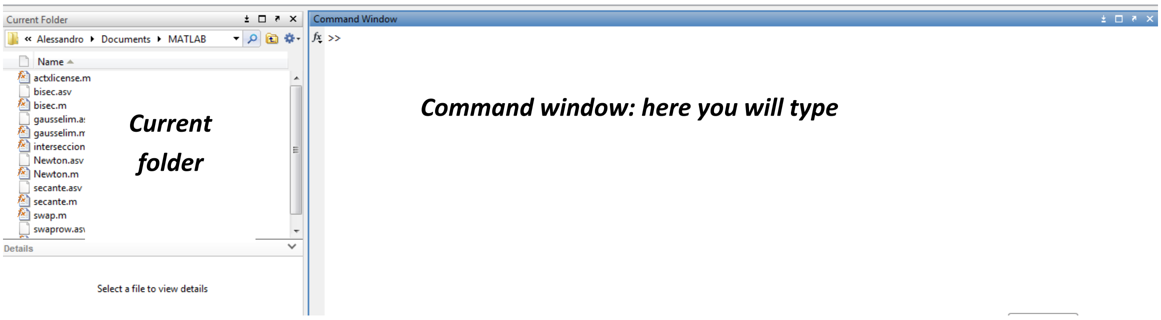

Change the current folder to the "MatLab_Scripts" folder within the Ion Torrent folder.

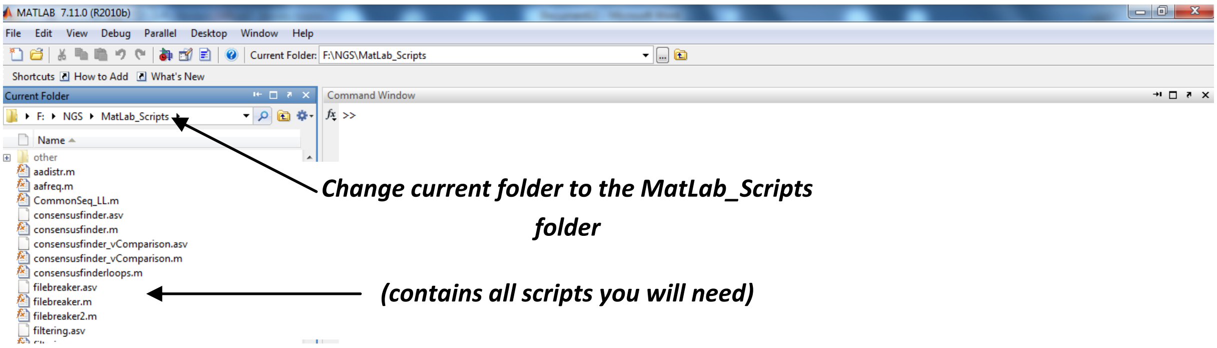

2. Step1: separating files by barcodes

Function information

Starting with the data file with all the sequences (.fastq), Step1 will generate files containing reads according to the barcodes. Running time depends on the size of the file, it takes between 15 and 20 minutes for a 500Mb file in a standard laptop computer.

Running the function

Type "Step1" in the command window:

```
>> Step1
```

A dialog box will open that allows you to choose the file (the starting file in .fastq format).

While running you will see:

```
>> Step1

BC =

  Columns 1 through 10

    'GCATAG'    'CGTATC'    'ATCGCA'    'ACGATA'    'AGACTC'    'GATACA'    'CATCTC'    'GTTCAG'    'TACCAG'    'ATGGAG'

  Columns 11 through 12

    'AGTTAC'    'GGTGAA'

Condireing barcodes with perfect match
Chip-specific code = @ST6XR
Number of barcodes = 12
reading file...
reading file...
reading file...
reading file...
reading file...
reading file...
reading file...
reading file...
reading file...
reading file...
reading file...
reading file...
reading file...
reading file...
File read in 690.7334 sec
Sorting by barcode...
Sorting by barcode...
Sorting by barcode...
Sorting by barcode...
Sorting by barcode...
Sorting by barcode...
Sorting by barcode...
Sorting by barcode...
Sorting by barcode...
Barcodes asigned in 578.4936 sec
```

Barcodes used. If not barcode is specified the 12 barcodes described in this publication are used. You can specify your own barcodes (see advanced options).

Only reads with perfect barcodes will be considered. You can optionally allow one insertion, deletion or mutation in the barcode (see advanced options).

Chip-specific code: for information, this is the code of the chip in the fastq file.

Step1 takes several minutes, and it indicates the action is performing (reading the file or sorting by barcode). This time is for a 316™ chip.

Output

Once it has finished, a new folder appears within the folder where the initial filename.fastq file was, called "filename_BC". Inside, there are a series of files called "BC1.txt", "BC2.txt", "BC3.txt", etc. containing the reads that corresponded to barcodes 1, 2 3, etc.

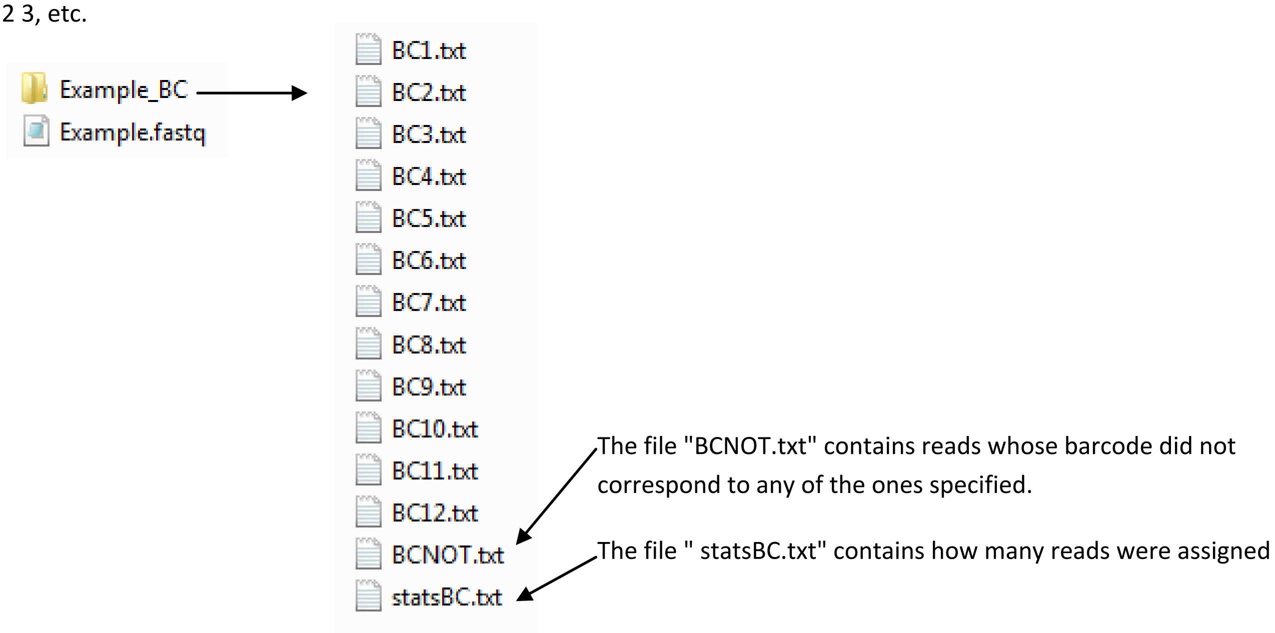

This is how each file looks like:

```
GCATAGTTTCTATGCGGCCCAGCCGGCCATGGCAGCGTGTTAGGATGATCCGGATGGGTGCTTGGGTTGTCTGACGGGGTGTGGCGGTTCTGGCGCTGAAAC  ?B@<<7996;A?AC??
=@D@EED@C::8<CCCCCCCCAAC@AA?BCCDCC>A=CECCACCCC@CDAC@BBBD@@9999-9DAA@DEAC@CBB@A@@?99909
GCATAGTTTCTATGCGGCCCAGCCGGCCATGGCAGCGTGTCCGCTGTCGACGGATGGAGTCGCTGTTTTAGCTGGGCCTTTTTTGTGGTCGGTTCTTG  143.../6546<AB?@?
AC>BBB7;;<=ABDBCCCC;;9?=<4949994449:44484444944//8806/////+/,/88::+;8656//,/7,,**
GCATAGTTTCTATGCGGCCCAGCCGGCCATGGCAGCGTGTGAGTAGGATACGGTTCCGTGCACGCTGCATCTGCTGGGTGTGGCGGTTCTGGCGCTGAAAC  >BBB>>@B?
BBABBBCDBC<@@A596;7<<CBCCCCCCBCCCCCCCACB?@@?=<??;;<CFBCCCCCCCC;;;>>>:>@AA>BC?B@BCC?BAABAAA>B
GCATAGTTTCTATGCGGCCCAGCCGGCCATGGCAGCGTGTTGAGC  >A????AA??BBBBBB?AA=BBF@B=@=?BBCCBA>;7,()))((
GCATAGTTTCTATGCGGCCCAGCCGGCCATGGCAGCGTGTAAGCTGCGGCAGGGTAGTTGCAGGGAGGGGAGTTTTAATTGTGGCGGTTCTGGCGCTGAAAC  ??>?ADBC>@?>ABHC?
CC>A@@=@5459498=BCBBBBBC@CHCB:::<CCC?CCECBCG<;;;<CDHA<;;CC8=>B@CCC@CC@C@CCC@CCA@ACE@C
GCATAGTTTCTATGCGGCCCAGCCGGCCATGGCAGCGTGTGCGGGTACGG  <=CECBAA;<<?A@BA@BB>@A;248<CACDBCCCB876/1,,,*,,,,,*
GCATAGTTTCTATGCGGCCCAGCCGGCCATGGCAGCGTGTGGTTGATGGTT  :<C@<<?><@ABCCBB@AAACB@6989>AABACCCBFB@@@59694/2*,*
GCATAGTTTCTATGCGGCCCAGCCGGCCATGGCAGCGTGTTGGGAG  ?BCBA?AA=@BBBBBB@BC?@>>2459599DCCCBB;:22*,,+*(
GCATAGTTTCTATGCGGCCCAGCCGGCCATGGCAGCGTGTGGAGTGTGGGTGGAA  7<BA?ABB=AABBB@?>?C>BBC:::;;>BBC@CDCCAAACC@<;;-,,,*,2*,*
GCATAGTTTCTATGCGGCCCAGCCGGCCATGGCAGCGTGTGGTGCGTTAGGTGG  9;@>97999:>BBCBB@BB>BC@697;79CBABBBCCB;;<,1,,,,*,,*,/8
GCATAGTTTCTATGCGGCCCAGCCGGCCATGGCAGCGTGTGTGGGTGTGGATAGGGATTGCCGTTAGGGGAAGGATCTGTGTGGCGGTTCTGGCGCTGAAAC  6;AA;?
BC>CABCCCB@DB>BBB?C>@@ADDCCCCBCCCDECCC>CBBB@BBCCIBBB:;<BBA>BBAA7B>B?CCBBBBBBB=BB=@=@CC@CEBB@AA<A
GCATAGTTTCTATGCGGCCCAGCCGGCCATGGCAGCGTGTAAGCGGATGGGTTCTGGTTGCGGGTTTGCTGGGTGGATGTGTGGCGGTTCTGGCGCTGAAAC
@BBBAACB>B88<CGDACC?CBA697;7<@CADCCBCBBGC@BCB>@:::-9<>?@=@>@BCH?CHCCDCCGBAG?>>>113>;>>@D@CAB@BCDCBCC@C
GCATAGTTTCTATGCGGCCCAGCCGGCCATGGCAGCGTGTTACTGGA  ?BCBB?AA??BAAABB>BC>A==698;;AADBECCC;872*****)(
GCATAGTTTCTATGCGGCCCAGCCGGCCATGGCAGCGTGTGACGTAT  ?BBBB?AA???>@===@AA>BBC>@>@>AADABB@BC;:,,*****(
GCATAGTTTCTATGCGGCCCAGCCGGCCATGGCAGCGTGTATGGATTGGCATGGTTTGTGCGTTGGGAATGGGTAGATTTGTGGCGGTTCTGGCGCTGAAAC  5;??@BBE?BCABB@?
>>>;;;>,1-1:<ABADHCCAAACCCCBBCCB@AAACCFH>CBCEBB8<;79@@@B=@A@9;;9;AB?BB?C?BBBBBBAB???6=
GCATAGTTTCTATGCGGCCCAGCCGGCCATGGCAGCGTGTAGAGGTGTTAG  =@A?;@AC=;B9::BB?BCABCB@B7:29<@@CABBCBAA;111-1,,2,,
GCATAGTTTCTATGCGGCCCAGCCGGCCATGGCAGCGTGTCTTGGTGCGGATGAGTATTGCTTGTTTGGTTAGCTTGATTGTGGCGGTTCTGGCGCTGAAAC  9<CCAA@B>BBABBCC?
BB::99296959?EACCCCCBCCBB>?=?B?=>@CBCBCCC?BGD>AAB<@7<;<CDI?BBB@CEC@CC@C@DCC@@@>>;;:6;
```

Advanced options

There are optional input parameters:

- **Using different barcodes:** different barcodes can be specified in the input (there is no limit on the number of barcodes but they must all have the same length). They can be specified as follows:

```
>> Step1('bc',{'GCAT','CGTA','ATCG'})

BC =

    'GCAT'    'CGTA'    'ATCG'

Condireing barcodes with perfect match
Chip-specific code = @R5U21
Number of barcodes = 3
reading file...
File read in 36.1843 sec
Sorting by barcode...
Barcodes asigned in 2.9565 sec
```

'bc' indicates that what comes next are the barcodes

Barcodes must be separated by comma, in single braket and within { }

Times for "Example.fastq" file

- **Allowing one insertion, mutation or deletion in the barcodes:** optionally, reads with one insertion, mutation or deletion in the barcode can be rescued with this option. It can be indicated as follows:

```
>> Step1('indelmut','on')

BC =

  Columns 1 through 9

    'GCATAG'    'CGTATC'    'ATCGCA'    'ACGATA'    'AGACTC'    'GATACA'    'CATCTC'    'GTTCAG'    'TACCAG'

  Columns 10 through 12

    'ATGGAG'    'AGTTAC'    'GGTGAA'

Considering barcodes having 1 in-del-mut
Chip-specific code = @R5U21
Number of barcodes = 12
reading file...
File read in 29.496 sec
Sorting by barcode...
Barcodes asigned in 11.208 sec
```

- Several inputs can be combined as follows:
  `Step1('indelmut','on', 'bc',{'GCAT','CGTA','ATCG'})`
  `Step1('bc',{'GCAT','CGTA','ATCG'},'indelmut','on')`

  The order of the inputs is not relevant as long as the input name (e.g. "indelmut") and its value (e.g. "on") come one after the other:

```
>> Step1('indelmut','on','bc',{'GCAT','CGTA','ATCG'})

BC =

    'GCAT'    'CGTA'    'ATCG'

Considering barcodes having 1 in-del-mut
Chip-specific code = @R5U21
Number of barcodes = 3
reading file...
File read in 37.3557 sec
Sorting by barcode...
Barcodes asigned in 3.6556 sec
```

3. Step2: quality filtering and translation

Function information

In this step you will analyze the reads corresponding to your barcode of interest. Low quality reads will be removed and sequences will be translated and sorted by abundance. Amber codons are translated to glutamine. Optionally, it can correct certain sequencing errors.

Running the function

Type "Step2" in the command window:

```
>> Step2
```

A dialog box opens that allows you to choose the file (one of the "BCn.txt" outputs of *Step1*).

While running, you will see:

```
>> Step2
Maximum number of bases below quality accepted = 3
Quality threshold = 18 , meaning p = 0.0158
Peptide start not specified, using default start ATGGC
Peptide end not specified, using default end GCTGAAAC
No minimum length of the peptide
No maximum length of the peptide
No intermediate limit
No fixing sequencing errors
Quality filter completed in 0.32906 sec
Translation completed in 0.46944 sec
```

These are default quality filter parameters: 3 bases below Q18. You can change these settings (see advanced options).

You can specify the nucleotide sequence of the start and the end of the region of interest. <u>If you are not using bicyclic peptide libraries you must change these default values</u> (see advanced options).

You can specify a minimum, intermediate and maximum length of the peptide (residues within the start and the end of the previously specified region). See advanced options.

It indicates the time taken in processing the quality filter and the translation respectively.

Optionally you can fix sequencing errors (only recommended when a few super-abundant clones dominate the population). See advanced options.

Output

A new folder appears within the input folder, called "Translation_BCn". It contains a file called "Translated_BCn_GOOD.txt" containing the translated good quality reads sorted by abundance. It also contains a file called "Translated_BCn_stats.txt" showing the total number of reads, the total number of different sequences and the abundance of the most frequent sequence.

"Translated_BC3_GOOD.txt" (from initial Example.fastq):

peptide seq.	abun.	nucleotide seq.
MAACTQSACSARVVCGGSG	73	ATGGCAGCATGCACGTAGTCTGCTTGCTCGGCGAGGGTTGTGTGTGGCGGTTCTGGCG
MAACAPDQCTKFTMCGGSG	33	ATGGCAGCATGCGCTCCGGATCAGTGCACTAAGTTTACTATGTGTGGCGGTTCTGGCG
MAACTYALCTARTFCGGSG	25	ATGGCAGCATGCACTTATGCTCTGTGCACTGCGCGTACGTTTTGTGGCGGTTCTGGCG
MAACSASQCSARIGCGGSG	21	ATGGCAGCATGCTCTGCTTCGTAGTGCTCTGCTAGGATTGGTTGTGGCGGTTCTGGCG
MAACKHSDCTARFPCGGSG	19	ATGGCAGCATGCAAGCATAGTGATTGCACTGCTCGGTTTCCTTGTGGCGGTTCTGGCG
MAACWTPTCSARSHCGGSG	19	ATGGCAGCATGCTGGACGCCTACGTGCTCTGCTCGTTCGCATTGTGGCGGTTCTGGCG
MAACPVKPSCHSGRCGGSG	18	ATGGCAGCATGCCCTGTTAAGCCGTCTTGCCATTCTGGGAGGTGTGGCGGTTCTGGCG
MAACAQATSCQTARCGGSG	18	ATGGCAGCATGCGCGTAGGCGACTTCGTGCTAGACTGCGCGTTGTGGCGGTTCTGGCG
MAACFRYQCTARSHCGGSG	16	ATGGCAGCATGCTTTCGTTATCAGTGCACGGCGCGTTCTCATTGTGGCGGTTCTGGCG
MAACPLSACSGRTLCGGSG	15	ATGGCAGCATGCCCGCTTTCTGCGTGCTCGGGGAGGACGTTGTGTGGCGGTTCTGGCG
MAACMLSGSCTARSCGGSG	14	ATGGCAGCATGCATGTTGTCTGGTTCTTGCACGGCTAGGTCGTGTGGCGGTTCTGGCG

Advanced options

- **Changing default quality parameters:** the quality filter removes all reads that contain a certain number of bases below a certain quality threshold. The default values for these two parameters are 3 bases below Q18. You can modify these values in the input as follows: `Step2('q',n,'badmax',m)`
  Where "n" is 18 for Q18, 20 for Q20, etc. And "m" is the maximum number of bases below that threshold allowed.
  e.g:

```
>> Step2('q',20,'badmax',4)
Maximum number of bases below quality accepted = 4
Quality threshold = 20 , meaning p = 0.01
Peptide start not specified, using default start ATGGC
Peptide end not specified, using default end GCTGAAAC
No minimum length of the peptide
No maximum length of the peptide
No intermediate limit
No fixing sequencing errors
Quality filter completed in 0.11192 sec
Translation completed in 0.0027165 sec
```

- **Changing peptide start and end:** Step2 evaluates the quality of a specified region. Default values for peptide start and end are only valid for bicyclic peptide libraries:

```
barcode      annealing region
           F   Y   A   A   Q   P   A   M   A   X   C    X      C   X   G   S   G   A   E
GCATAG T TTC TAT GCG GCC CAG CCG GCC ATG GCA NNK TGC (NNK)n TGT NNK GGT TCT GGC GCT GAA C
```

start of the peptide for bicyclic peptide libraries: ATGGC
end of the peptide for bicyclic peptide libraries: GCTGAAAC

You must change these values if you are using other libraries. You need to specify a constant nucleotide region before and after the random region of interest (can be the annealing regions of the primers). Requisites:
--> The "start" must begin with the first letter of a codon for the translation to be in frame.
--> The "end" should be long enough to minimize the chances that it appears in the random region. In any case, if the "end" sequence is found twice (or more) in a read, it will consider the last one as the end of the peptide. However, artifacts can appear if the true "end" sequence is mutated and it appears in the random region, since then it will consider the "end" sequence in the random region as the end of the peptide.

You can change these values in the input as follows: `Step2('start','AAAAAA','end','TTTTTT')`, e.g:

```
>> Step2('start','GCATGC','end','TGTGGC')
Maximum number of bases below quality accepted = 3
Quality threshold = 18 , meaning p = 0.0158
Peptide start specifcied: GCATGC
Peptide end specified: TGTGGC
No minimum length of the peptide
No maximum length of the peptide
No intermediate limit
No fixing sequencing errors
Quality filter completed in 0.28082 sec
Translation completed in 0.48225 sec
```

- **Specifying a minimum, maximum or intermediate length of the peptide:** once the start and the end of the peptide region have been specified, peptides longer or shorter than expected can be filtered out. An intermediate length can be also used to divide the output in two files containing peptides longer or shorter than that length.
  You can specify it as follows: `Step2('uplimit',m,'downlimit',n,'midlimit',o)`
  Where "m" is the maximum length of the peptide (in residues), "n" the minimum and "o" the intermediate.

  For example, to consider only peptides whose length is between 5 and 25 residues:

```
>> Step2('uplimit',25,'downlimit',5)
Maximum number of bases below quality accepted = 3
Quality threshold = 18 , meaning p = 0.0158
Peptide start not specified, using default start ATGGC
Peptide end not specified, using default end GCTGAAAC
Minimum length of the peptide in bases: 14
Maximum length of the peptide in bases: 76
No intermediate limit
No fixing sequencing errors
Quality filter completed in 0.29421 sec
Translation completed in 0.61337 sec
```

  To divide the output in peptides in two files: one with peptides longer than 12 residues, and the other with peptides of 12 residues or shorter::

```
>> Step2('midlimit',12)
Maximum number of bases below quality accepted = 3
Quality threshold = 18 , meaning p = 0.0158
Peptide start not specified, using default start ATGGC
Peptide end not specified, using default end GCTGAAAC
No minimum length of the peptide
No maximum length of the peptide
Intermediate limit in bases (<=): 37
No fixing sequencing errors
Quality filter completed in 0.28456 sec
Translation completed in 0.57042 sec
Translation completed in 0.0031404 sec
```

  Two "translation" files have been generated:
  Translated_BCn_longGOOD.txt
  Translated_BCn_shortGOOD.txt

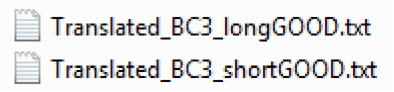

  You can combine all inputs as long as the pairs 'inputname','inputvalue' come together:

```
>> Step2('midlimit',12,'start','ATGGC','q',19)
Maximum number of bases below quality accepted = 3
Quality threshold = 19 , meaning p = 0.0126
Peptide start specifcied: ATGGC
Peptide end not specified, using default end GCTGAAAC
No minimum length of the peptide
No maximum length of the peptide
Intermediate limit in bases (<=): 37
No fixing sequencing errors
Quality filter completed in 0.27928 sec
Translation completed in 0.51037 sec
Translation completed in 0.0049327 sec
```

- **Correcting sequencing errors:** sequencing errors leading to insertion, deletion or mutation variants of very high abundant peptides can be optionally corrected. You can run this option either within Step2.m (as described here), or run before Step2 without this option, evaluate the output, and then choose to run fixingerrors.m.

This option will start with the most abundant sequence, and look for nucleotide sequences that only differ in one or two positions in the rest of the dataset. It will merge them with the most abundant one. It will do so with the number of sequences specified:

```
Step2('fixerr',n)
```

Where "n" is the number of sequences to be corrected. For example, to merge all reads corresponding to the 5 most abundant clones:

```
>> Step2('fixerr',5)
Maximum number of bases below quality accepted = 3
Quality threshold = 18 , meaning p = 0.0158
Peptide start not specified, using default start ATGGC
Peptide end not specified, using default end GCTGAAAC
No minimum length of the peptide
No maximum length of the peptide
No intermediate limit
Fixing sequencing errors: merging sequences with only 5 differences in the DNA sequence
Quality filter completed in 0.28947 sec
Translation completed in 0.57274 sec
Fixing errors of top 5 abundant sequences
Looking for sequencing errors of sequence 1
Looking for sequencing errors of sequence 2
Looking for sequencing errors of sequence 3
Looking for sequencing errors of sequence 4
Looking for sequencing errors of sequence 5
Number of sequences before = 1612
Number of sequences after = 1612
Fixing completed in 1.6347 sec
```

Running time depends on the number of sequences it will "fix" and the number of different sequences in the dataset. When it has finished a sequence, it will indicate it in the command window, allowing to estimate the total running time.

- correctiondataTranslated_BC3_GOOD.txt
- fixerrTranslated_BC3_GOOD.txt
- Translated_BC3_GOOD.txt
- Translated_stats.txt

Within the "Translated_BCn" folder, an additional file appears called "fixerrTranslated_BCn_GOOD.txt". This contains the sequences after correcting sequencing errors. The file "Translated_BCn_GOOD.txt" contains the sequences before correcting sequencing errors:

Translated....txt

```
MAACTQSACSARVVCGGSG    73  ATGGCAGCATGCACGTAGTCTGCTTGCTCGGCGAGGGTTGTGTGTGGCGGTTCTGGCG
MAACAPDQCTKFTMCGGSG    33  ATGGCAGCATGCGCTCCGGATCAGTGCACTAAGTTTACTATGTGTGGCGGTTCTGGCG
MAACTYALCTARTFCGGSG    25  ATGGCAGCATGCACTTATGCTCTGTGCACTGCGCGTACGTTTTGTGGCGGTTCTGGCG
MAACSASQCSARIGCGGSG    21  ATGGCAGCATGCTCTGCTTCGTAGTGCTCTGCTAGGATTGGTTGTGGCGGTTCTGGCG
MAACKHSDCTARFPCGGSG    19  ATGGCAGCATGCAAGCATAGTGATTGCACTGCTCGGTTTCCTTGTGGCGGTTCTGGCG
```

fixerrTranslated....txt

```
MAACTQSACSARVVCGGSG    76  ATGGCAGCATGCACGTAGTCTGCTTGCTCGGCGAGGGTTGTGTGTGGCGGTTCTGGCG
MAACAPDQCTKFTMCGGSG    34  ATGGCAGCATGCGCTCCGGATCAGTGCACTAAGTTTACTATGTGTGGCGGTTCTGGCG
MAACTYALCTARTFCGGSG    27  ATGGCAGCATGCACTTATGCTCTGTGCACTGCGCGTACGTTTTGTGGCGGTTCTGGCG
MAACSASQCSARIGCGGSG    21  ATGGCAGCATGCTCTGCTTCGTAGTGCTCTGCTAGGATTGGTTGTGGCGGTTCTGGCG
MAACKHSDCTARFPCGGSG    20  ATGGCAGCATGCAAGCATAGTGATTGCACTGCTCGGTTTCCTTGTGGCGGTTCTGGCG
```

In this case, there were 3 reads in the dataset that only differed in one or two positions from the most abundant clone. They were merged together and therefore the abundance went from 73 to 76.

A new folder appears called "correctiondata..." containing: a file with the error rates of all sequences corrected, and two files where all mergings are specified. This folder is just for information.

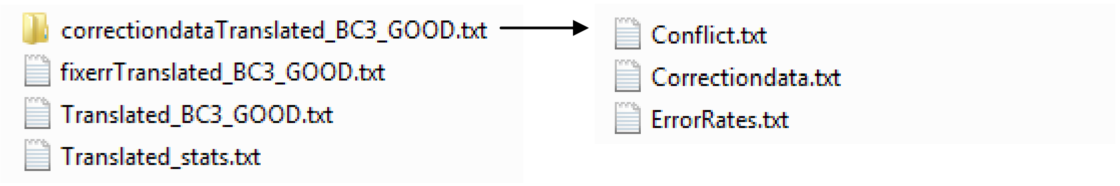

ErrorRates.txt

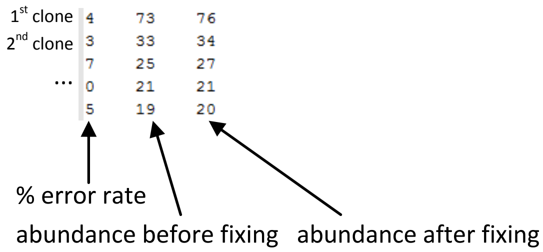

Correctiondata.txt (here showing three merging events for the most abundant clone)

```
73  ATGGCAGCATGCACGTAGTCTGCTTGCTCGGCGAGGGTTGTGTGTGGCGGTTCTGGCG   MAACTQSACSARVVCGGSG
1   ATGGCAGCATGCACGTAGTCTGCTTGCTCGGCGAGGGTTGTGTGTGGCGGTTCTAGCG   MAACTQSACSARVVCGGSS
1

74  ATGGCAGCATGCACGTAGTCTGCTTGCTCGGCGAGGGTTGTGTGTGGCGGTTCTGGCG   MAACTQSACSARVVCGGSG
1   ATGGCAGCATGCACGTAGTCTGCTTGCTCGGCGAGGGTTGTGTGTGGCGGTTCTGCG   MAACTQSACSARVVCGGSA
1

75  ATGGCAGCATGCACGTAGTCTGCTTGCTCGGCGAGGGTTGTGTGTGGCGGTTCTGGCG   MAACTQSACSARVVCGGSG
1   ATGGCAGTATGCACGTAGTCTGCTTGCTCGGCGAGGGTTGTGTGTGGCGGTTCTGGCG   MAVCTQSACSARVVCGGSG
1
```

Conflict.txt has potential problematic merging events (where the difference in abundance is lower than 4x)

```
4  ATGGCAGCATGCATTTTTTTATAAGTCTTGCAAGTATTCGTTGTGTGGCGGTTCTGGCG   MAACIFL*VLQVFVVWRFW
3   ATGGCAGCATGCATTTTTTATAAGTCTTGCAAGTATTCGTTGTGTGGCGGTTCTGGCG   MAACIFYKSCKYSLCGGSG
1
```

- **Getting more information: intermediate quality files and translation of bad quality sequences and too long / too short sequences:** optionally, all quality files and their translation can be kept indicating it in the input as follows:
  `Step2('keepqf','on')`
  `Step2('translateall','on')`
  Or both combined:
  `Step2('keepqf','on','translateall','on')`

  - Quality files appear in a folder called "QF_BCn" within the same folder as the input "BCn.txt" file:

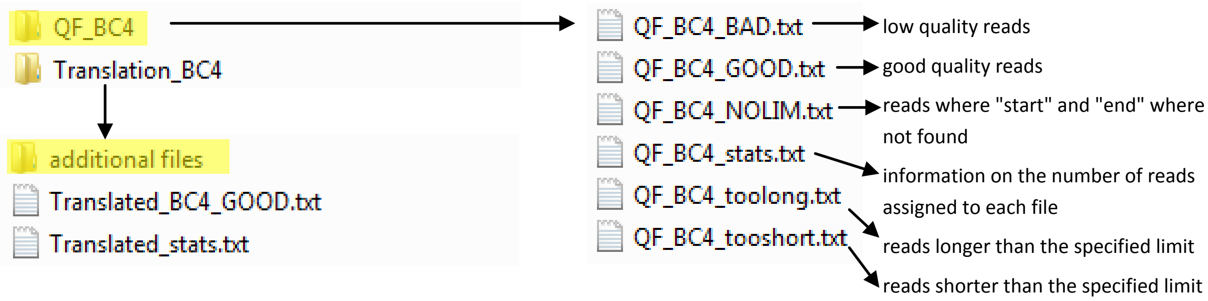

  - The translation of the bad quality and off-limits files are in a folder called "additional files" withint the Translation_BCn folder.

4. Clustering

Function information

This script compares a chosen number of sequences (if not specified, compares the top 200), and groups them into families that share higher sequence similarity. Even within a cluster, more similar sequences appear together.

Running the function

Type "Clustering" in the command window:

```
>> Clustering
```

A dialog box opens allowing you to choose the file. The input files accepted are the output of Step2 (Translated....txt and fixerrTranslated....txt), and also the outputs of LoopLengths and FindSeq (described later). The requisite is that the contents of the text file are in the format: peptide sequence – abundance - nucleotide sequence, e.g:

```
MAACTQSACSARVVCGGSG    76  ATGGCAGCATGCACGTAGTCTGCTTGCTCGGCGAGGGTTGTGTGGCGGTTCTGGCG
MAACAPDQCTKFTMCGGSG    34  ATGGCAGCATGCGCTCCGGATCAGTGCACTAAGTTTACTATGTGTGGCGGTTCTGGCG
MAACTYALCTARTFCGGSG    27  ATGGCAGCATGCACTTATGCTCTGTGCACTGCGCGTACGTTTTGTGGCGGTTCTGGCG
MAACSASQCSARIGCGGSG    21  ATGGCAGCATGCTCTGCTTCGTAGTGCTCTGCTAGGATTGGTTGTGGCGGTTCTGGCG
MAACKHSDCTARFPCGGSG    20  ATGGCAGCATGCAAGCATAGTGATTGCACTGCTCGGTTTCCTTGTGGCGGTTCTGGCG
MAACAQATSCQTARCGGSG    20  ATGGCAGCATGCGCGTAGGCGACTTCGTGCTAGACTGCGCGTTGTGGCGGTTCTGGCG
MAACWTPTCSARSHCGGSG    19  ATGGCAGCATGCTGGACGCCTACGTGCTCTGCTCGTTCGCATTGTGGCGGTTCTGGCG
MAACPVKPSCHSGRCGGSG    18  ATGGCAGCATGCCCTGTTAAGCCGTCTTGCCATTCTGGGAGGTGTGGCGGTTCTGGCG
MAACPVLPQCSARSCGGSG    18  ATGGCAGCATGCCCTGTGCTTCCTTAGTGCTCGGCGAGGTCTTGTGGCGGTTCTGGCG
MAACMLSGSCTARSCGGSG    17  ATGGCAGCATGCATGTTGTCTGGTTCTTGCACGGCTAGGTCGTGTGGCGGTTCTGGCG
```

When you launch the function, you will see:

```
>> Clustering
Comparing top 200 abundant sequences. To change, specify number_dif
Minimum cluster size = 3, (smaller clusters will be merged in "cluster mixed")
Stringency = 0.5
No c-ter specified.
Minimum abundance = 2
Number of total sequences considered = 1122
Number of different sequences = 219
```

Parameters that can be changed (**see advanced options for details**):

By default, it compares the 200 most abundant sequences.

Clusters containing 1 or 2 peptides only will be put together in the last cluster called "cluster mixed".

Default stringency is 0.5. If you are not satisfied with the groups, you can change this parameter.

Optionally, a C-terminal peptide sequence can be specified to discard frame-shifted clones.

Minimum abundance of the peptides considered.

At the end, it is comparing 219 sequences because the abundance of the 200th sequence was 2, and there were 19 more with the same abundance, which are also considered.

While running, the command window indicates which step of the clustering is being performed:

```
>> Clustering
Comparing top 200 abundant sequences. To change, specify number_dif
Minimum cluster size = 3, (smaller clusters will be merged in "cluster mixed")
Stringency = 0.5
No c-ter specified.
Minimum abundance = 2
Number of total sequences considered = 1122
Number of different sequences = 219
Distance calculated in 19.7294 sec
Tree done in 0.039675 sec
Clustering done in 7.9756 sec
Sequences remaining for the second clustering = 102
Distance calculated in 4.0768 sec
Tree done in 0.022349 sec
Clustering done in 1.4603 sec
total time = 33.3336 sec
```

A first round of clustering is performed.

Then the sequences in the "cluster mixed" are re-taken for a second round of clustering

Output

A new file appears within the folder where there was the initial file, named "Clusters_....txt", and a series of picture files "Logo_GroupN.jpg" corresponding to the sequence logos of each of the groups (this option can be disabled in the input -> `Clustering(...,'logos','off')`).

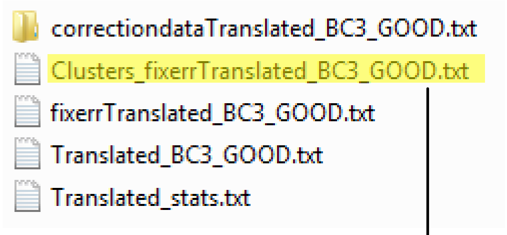


```
Group1
MAACGVVTCTARQHCGGSG     11    ATGGCAGCATGCGGTGTTGTGACGTGCACGGCTCGTTAGCATTGTGGCGGTTCTGGCG
MAACGIANCTARAQCGGSG     3     ATGGCAGCATGCGGGATTGCTAATTGCACGGCGCGTGCTTAGTGTGGCGGTTCTGGCG
MAACMQHRCSARTGCGGSG     3     ATGGCAGCATGCATGTAGCATAGGTGCTCGGCGCGGACTGGTTGTGGCGGTTCTGGCG
MAACVQLRCTARTHCGGSG     3     ATGGCAGCATGCGTTCAGCTGCGTTGCACTGCTCGTACTCATTGTGGCGGTTCTGGCG
MAACRQSTCSARTYCGGSG     12    ATGGCAGCATGCCGGTAGTCTACTTGCTCTGCTAGGACGTATTGTGGCGGTTCTGGCG
MAACKQSVCTARTLCGGSG     6     ATGGCAGCATGCAAGTAGAGTGTGTGCACGGCTAGGACGTTGTGTGGCGGTTCTGGCG
MAACTQSACSARVVCGGSG     76    ATGGCAGCATGCACGTAGTCTGCTTGCTCGGCGAGGGTTGTGTGTGGCGGTTCTGGCG
MAACNESVCSARKQCGGSG     4     ATGGCAGCATGCAATGAGTCGGTGTGCTCTGCGCGTAAGTAGTGTGGCGGTTCTGGCG
MAACTYALCTARTFCGGSG     27    ATGGCAGCATGCACTTATGCTCTGTGCACTGCGCGTACGTTTTGTGGCGGTTCTGGCG
MAACSVSFCSARSFCGGSG     5     ATGGCAGCATGCAGTGTGTCTTTTTGCTCTGCGCGGTCTTTTTGTGGCGGTTCTGGCG
MAACSLASCSARMLCGGSG     4     ATGGCAGCATGCTCTCTGGCGAGTTGCTCGGCGCGTATGTTGTGTGGCGGTTCTGGCG
MAACTLGNCTARAICGGSG     2     ATGGCAGCATGCACTCTTGGTAATTGCACGGCGAGGGCTATTTGTGGCGGTTCTGGCG
MAACPLSACSGRTLCGGSG     15    ATGGCAGCATGCCCGCTTTCTGCGTGCTCGGGGAGGACGTTGTGTGGCGGTTCTGGCG
MAACVISTCSARHDCGGSG     3     ATGGCAGCATGCGTTATTAGTACGTGCAGTGCGCGGCATGATTGTGGCGGTTCTGGCG
MAACVVSVCSARRSCGGSG     2     ATGGCAGCATGCGTGGTTAGTGTTTGCTCGGCGAGGCGGTCGTGTGGCGGTTCTGGCG
MAACRTAVCTARLLCGGSG     5     ATGGCAGCATGCCGTACTGCTGTGTGCACTGCTCGTTTGTTGTGTGGCGGTTCTGGCG
MAACATGVCTARLQCGGSG     2     ATGGCAGCATGCGCTACTGGTGTTTGCACGGCGCGTCTGCAGTGTGGCGGTTCTGGCG
MAACAASVCTARLFCGGSG     10    ATGGCAGCATGCGCGGCTTCGGTGTGCACTGCTAGGTTGTTTTGTGGCGGTTCTGGCG
MAACSAAYCTARLQCGGSG     3     ATGGCAGCATGCAGTGCGGCTTATTGCACGGCTAGGCTGTAGTGTGGCGGTTCTGGCG
MAACKLSVCTSRLTCGGSG     2     ATGGCAGCATGCAAGTTGAGTGTTTGCACGTCGAGGCTTACTTGTGGCGGTTCTGGCG
MAACKETQCTARITCGGSG     4     ATGGCAGCATGCAAGGAGACGTAGTGCACGGCGCGGATTACTTGTGGCGGTTCTGGCG
MAACFNTQCTARLSCGGSG     2     ATGGCAGCATGCTTTAATACGTAGTGCACTGCGCGTCTTTCTTGTGGCGGTTCTGGCG
MAACQNSSCTARLVCGGSG     2     ATGGCAGCATGCTAGAATAGTTCTTGCACTGCTCGTTTGGTTTGTGGCGGTTCTGGCG
MAACSSDNCTARVTCGGSG     3     ATGGCAGCATGCTCGTCTGATAATTGCACGGCTAGGGTTACTTGTGGCGGTTCTGGCG
MAACSTPNCTARLRCGGSG     2     ATGGCAGCATGCTCTACTCCGAATTGCACTGCTAGGTTGCGTTGTGGCGGTTCTGGCG
MAACSTPQCTARWVCGGSG     3     ATGGCAGCATGCAGTACTCCTTAGTGCACTGCTCGGTGGGTTTGTGGCGGTTCTGGCG
MAACSASQCSARIGCGGSG     21    ATGGCAGCATGCTCTGCTTCGTAGTGCTCTGCTAGGATTGGTTGTGGCGGTTCTGGCG
MAACSPSQCTARAGCGGSG     11    ATGGCAGCATGCTCTCCTTCGTAGTGCACTGCTCGTGCGGGGTGTGGCGGTTCTGGCG
MAACSLRLCTARIGCGGSG     2     ATGGCAGCATGCTCGTTGCGGCTTTGCACGGCTCGTATTGGTTGTGGCGGTTCTGGCG
MAACPLQLCTARYPCGGSG     14    ATGGCAGCATGCCCTTTGTAGTTGTGCACGGCTCGGTATCCTTGTGGCGGTTCTGGCG
MAACSLSLCSARYPCGGSG     5     ATGGCAGCATGCTCTCTGAGTCTTTGCTCTGCGCGTTATCCGTGTGGCGGTTCTGGCG
MAACTFSICSARLPCGGSG     2     ATGGCAGCATGCACGTTTTCTATTTGCTCTGCGAGGTTGCCTTGTGGCGGTTCTGGCG

Group2
MAACLSSARCGSIACGGSG     2     ATGGCAGCATGCCTTTCTAGTGCGCGTTGCGGTTCTATTGCGTGTGGCGGTTCTGGCG
MAACLSTARCTFTQCGGSG     3     ATGGCAGCATGCCTTTCGACTGCTCGGTGCACGTTTACGCAGTGTGGCGGTTCTGGCG
MAACHATARCLFASCGGSG     2     ATGGCAGCATGCCATGCGACGGCTCGTTGCTTGTTTGCGTCGTGTGGCGGTTCTGGCG
MAACSSTARCELSYCGGSG     3     ATGGCAGCATGCTCGTCTACTGCGCGTTGCGAGCTTTCGTATTGTGGCGGTTCTGGCG
MAACGGSARCHLSMCGGSG     2     ATGGCAGCATGCGGTGGTTCGGCGCGTTGCCATCTTTCTATGTGTGGCGGTTCTGGCG
```

Advanced options

- **Changing the number of sequences to compare:** there are two options, either you choose the number of different sequences to compare, or you choose the minimum abundance.

  In the first case, specify the input 'number_dif' as follows:
  `Clustering('number_dif',n)`
  Where "n" is the number of different sequences you want to compare. For example to compare the 1000 most abundant sequences:

```
>> Clustering('number_dif',1000)
Comparing top 1000 abundant sequences.
```

  In the second case, specify the 'min_abun' as follows:
  `Clustering('min_abun',m)`
  Where "m" is the minimum abundance of the peptides to be considered. For example to compare all sequences whose abundance is 50 or higher:

```
>> Clustering('min_abun',50)
Comparing sequences whose minimum abundance is 50
```

- **Changing the minimum cluster size:** by default, clusters containing only one or two peptides are merged together in the last cluster called "consensus mixed". You can change this parameter as follows:
  `Clustering('min_clustersize',n)`
  For example, to accept only clusters whose size is higher than 5 peptides:

```
>> Clustering('min_clustersize',5)
Comparing top 200 abundant sequences. To change, specify number_dif
Minimum cluster size = 5, (smaller clusters will be merged in "cluster mixed")
Stringency = 0.5
No c-ter specified.
Minimum abundance = 2
Number of total sequences considered = 1122
Number of different sequences = 219
Distance calculated in 21.4739 sec
Tree done in 0.043212 sec
Clustering done in 7.946 sec
Sequences remaining for the second clustering = 124
Distance calculated in 5.9446 sec
Tree done in 0.02242 sec
Clustering done in 2.1477 sec
total time = 37.6104 sec
```

  This will change the groups coming from the second round of clustering since there will be more peptides available for it.

- **Changing the "stringency" of the grouping:** if the groups with the default stringency are not satisfying, increase or decrease this value (0 < stringency < 1). In general, higher stringency values will lead to less peptides in the "consensus mixed" and lower values will move some of the peptides of "consensus mixed" into the groups:

- **Specifying a constant C-terminal peptide sequence to remove frame-shifted clones:** in order to not consider frame-shifted clones, a constant C-terminal peptide sequence can be indicated, so only peptides having it will be taken. For example, for bicyclic peptide libraries and the "start" and "end" of the peptide used by default, 'GGSG' can be used:

```
>> Clustering('cter','GGSG')
Comparing top 200 abundant sequences. To change, specify number_dif
Minimum cluster size = 3, (smaller clusters will be merged in "cluster mixed")
Stringency = 0.5
Considering only sequences containing: GGSG.
```

5. LoopLengths (for cys-constrained monocyclic or bicyclic peptides)

Function information

This script separates sequences in different files according to the peptide format (i.e. number of cysteine residues and number of residues between them).

Running the function

Type LoopLengths in the command window:

```
>> LoopLengths
```

A dialog box opens that allows to choose the file. The outputs of Step2 ("Translated...txt" and "fixerrTranslated...txt" can be used as input of LoopLengths, as well as the outputs of FindSeq). The requisite is that the contents of the text file are in the format: peptide sequence – abundance - nucleotide sequence, e.g:

```
MAACTQSACSARVVCGGSG    76  ATGGCAGCATGCACGTAGTCTGCTTGCTCGGCGAGGGTTGTGTGGCGGTTCTGGCG
MAACAPDQCTKFTMCGGSG    34  ATGGCAGCATGCGCTCCGGATCAGTGCACTAAGTTTACTATGTGTGGCGGTTCTGGCG
MAACTYALCTARTFCGGSG    27  ATGGCAGCATGCACTTATGCTCTGTGCACTGCGCGTACGTTTTGTGGCGGTTCTGGCG
MAACSASQCSARIGCGGSG    21  ATGGCAGCATGCTCTGCTTCGTAGTGCTCTGCTAGGATTGGTTGTGGCGGTTCTGGCG
MAACKHSDCTARFPCGGSG    20  ATGGCAGCATGCAAGCATAGTGATTGCACTGCTCGGTTTCCTTGTGGCGGTTCTGGCG
MAACAQATSCQTARCGGSG    20  ATGGCAGCATGCGCGTAGGCGACTTCGTGCTAGACTGCGCGTTGTGGCGGTTCTGGCG
MAACWTPTCSARSHCGGSG    19  ATGGCAGCATGCTGGACGCCTACGTGCTCTGCTCGTTCGCATTGTGGCGGTTCTGGCG
MAACPVKPSCHSGRCGGSG    18  ATGGCAGCATGCCCTGTTAAGCCGTCTTGCCATTCTGGGAGGTGTGGCGGTTCTGGCG
MAACPVLPQCSARSCGGSG    18  ATGGCAGCATGCCCTGTGCTTCCTTAGTGCTCGGCGAGGTCTTGTGGCGGTTCTGGCG
MAACMLSGSCTARSCGGSG    17  ATGGCAGCATGCATGTTGTCTGGTTCTTGCACGGCTAGGTCGTGTGGCGGTTCTGGCG
```

After running the function:

```
>> LoopLengths
No C-terminus specified
No abundance cutoff applied
Total sequences considered = 1612
```

You can specify a C-terminal constant peptide sequence to discard frame-shifted clones, and/or a minimum abundance cutoff (see advanced options).

Total sequences considered (sum of abundances of the different sequences)

Output

A new folder is created called "LoopLengths_...", within the folder where the input file was, containing four sub-folders:

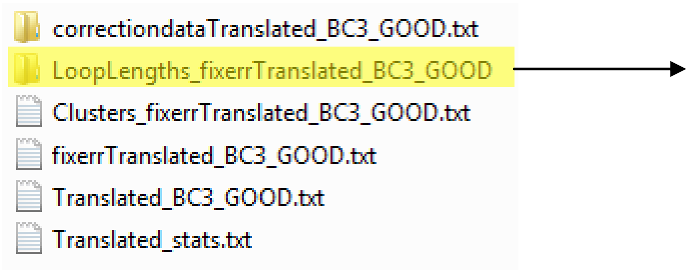

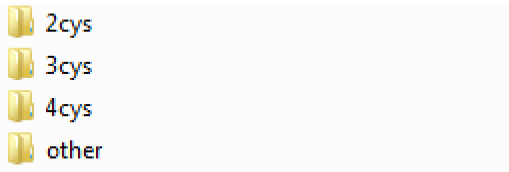

The folders contain the files corresponding to the different number of cysteines:

In the 2cys folder:

File	Contents
LoopLengths_3_twocys.txt	Peptides having two cysteines and 3 residues between them: C XXX C
LoopLengths_4_twocys.txt	Peptides having two cysteines and 4 residues between them: C XXXX C
LoopLengths_5_twocys.txt	etc.
LoopLengths_6_twocys.txt	
LoopLengths_8_twocys.txt	
LoopLengths_9_twocys.txt	
LoopLengths_10_twocys.txt	

In the 3cys folder:

File	Contents
LoopLengths_102_threecys.txt	Peptides having three cysteines "1x2": C X C XX C
LoopLengths_104_threecys.txt	Peptides having three cysteines "1x4": C X C XXXX C
LoopLengths_105_threecys.txt	
LoopLengths_7_threecys.txt	Peptides having three cysteines "0x7": C C XXXXXXX C
LoopLengths_600_threecys.txt	Peptides having three cysteines "6x0": C XXXXXX C C

In the 4cys folder:

File	Contents
LoopLengths_3_fourcys.txt	Peptides having four cysteines "0x0x3": C C C XXX C
LoopLengths_205_fourcys.txt	Peptides having four cysteines "0x2x5": C C XX C XXXXX C
LoopLengths_20203_fourcys.txt	Peptides having four cysteines "2x2x3": C XX C XX C XXX C

In the "other" folder:

File	Contents
LoopLengths_many_cys.txt	Peptides having more than four cysteines
LoopLengths_no_cys.txt	Peptides without cysteines
LoopLengths_one_cys.txt	Peptides with one cysteine
LoopLengths_stats.txt	Information: how many different and total sequences were assigned to each file

Advanced options

- **Specifying a constant C-terminal peptide sequence to remove frame-shifted clones:** in order to not consider frame-shifted clones, a constant C-terminal peptide sequence can be indicated, so only peptides having it will be taken. It must be specified in the input as follows:
  `LoopLengths('cter','XYZ')`
  where XYZ is the constant C-terminal peptide sequence. For example, for bicyclic peptide libraries and the "start" and "end" of the peptide used by default, 'GGSG' can be used:
  ```
  >> LoopLengths('cter','GGSG')
  ```
- **Specifying a minimum abundance cutoff:** it can be indicated in the input as follows:
  `LoopLengths('cutoff',n)`
  where "n" is the minimum abundance for a peptide to be considered. For example, to discard all peptides whose abundance is lower than 10:
  ```
  >> LoopLengths('cutoff',3)
  No C-terminus specified
  Considering sequences with a minimum abundance of 3
  ```

6. CommonSeq

Function information

Compares up to three different datasets and distributes common and exclusive sequences in different files.

Running the function

Type CommonSeq in the command window:

```
>> CommonSeq
```

A dialog box will open that allows you to choose the first file. The input files accepted are the output of Step2 (Translated....txt and fixerrTranslated....txt), and also the outputs of LoopLengths and FindSeq (described later). The requisite is that the contents of the text file are in the format: peptide sequence – abundance - nucleotide sequence, e.g:

```
MAACTQSACSARVVCGGSG    76  ATGGCAGCATGCACGTAGTCTGCTTGCTCGGCGAGGGTTGTGTGGCGGTTCTGGCG
MAACAPDQCTKFTMCGGSG    34  ATGGCAGCATGCGCTCCGGATCAGTGCACTAAGTTTACTATGTGGCGGTTCTGGCG
MAACTYALCTARTFCGGSG    27  ATGGCAGCATGCACTTATGCTCTGTGCACTGCGCGTACGTTTTGTGGCGGTTCTGGCG
MAACSASQCSARIGCGGSG    21  ATGGCAGCATGCTCTGCTTCGTAGTGCTCTGCTAGGATTGGTTGTGGCGGTTCTGGCG
MAACKHSDCTARFPCGGSG    20  ATGGCAGCATGCAAGCATAGTGATTGCACTGCTCGGTTTCCTTGTGGCGGTTCTGGCG
MAACAQATSCQTARCGGSG    20  ATGGCAGCATGCGCGTAGGCGACTTCGTGCTAGACTGCGCGTTGTGGCGGTTCTGGCG
MAACWTPTCSARSHCGGSG    19  ATGGCAGCATGCTGGACGCCTACGTGCTCTGCTCGTTCGCATTGTGGCGGTTCTGGCG
MAACPVKPSCHSGRCGGSG    18  ATGGCAGCATGCCCTGTTAAGCCGTCTTGCCATTCTGGGAGGTGTGGCGGTTCTGGCG
MAACPVLPQCSARSCGGSG    18  ATGGCAGCATGCCCTGTGCTTCCTTAGTGCTCGGCGAGGTCTTGTGGCGGTTCTGGCG
MAACMLSGSCTARSCGGSG    17  ATGGCAGCATGCATGTTGTCTGGTTCTTGCACGGCTAGGTCGTGTGGCGGTTCTGGCG
```

After choosing the first file a second dialog box will open allowing to choose the second file. After, a third dialog box will open allowing to choose the third file. If only two datasets need to be compared, press cancel in the third one.

After running the command window will display the number of different sequences and the sum of their abundances (total sequences) considered in each file:

```
>> CommonSeq
File 1: considering 7242 different sequences
File 1: considering 49636 total sequences
File 2: considering 19692 different sequences
File 2: considering 170538 total sequences
File 3: considering 709 different sequences
File 3: considering 1612 total sequences
Finished dataset 1
Finished dataset 2
Finished dataset 3
```

Output

A new folder "Comparison" **within the folder where the first file was** appears. Inside, there will be the following files

- Comparison_Seq1.txt, Comparison_Seq2.txt, Comparison_Seq3.txt → Contains sequences only found in dataset (file) 1, 2 or 3 respectively
- Comparison_Seq12.txt, Comparison_Seq13.txt, Comparison_Seq23.txt → Contains sequences found in 2 of the datasets
- Comparison_Seq123.txt → Contains sequences appearing in all 3 datastes
- Comparison_stats.txt → Information about how many different seqeunces and total reads are assigned to each file

Each output file is organised as follows (example from the Comparison_Seq12.txt file):

peptide sequence	abudance 1st	2nd	3rd	nucleotide sequence
MAACTQSACSARVVCGGSG	73	76	0	ATGGCAGCATGCACGTAGTCTGCTTGCTCGGCGAGGGTTGTGTGTGGCGGTTCTGGCG
MAACAPDQCTKFTMCGGSG	33	34	0	ATGGCAGCATGCGCTCCGGATCAGTGCACTAAGTTTACTATGTGTGGCGGTTCTGGCG
MAACTYALCTARTFCGGSG	25	27	0	ATGGCAGCATGCACTTATGCTCTGTGCACTGCGCGTACGTTTTGTGGCGGTTCTGGCG
MAACSASQCSARIGCGGSG	21	21	0	ATGGCAGCATGCTCTGCTTCGTAGTGCTCTGCTAGGATTGGTTGTGGCGGTTCTGGCG
MAACKHSDCTARFPCGGSG	19	20	0	ATGGCAGCATGCAAGCATAGTGATTGCACTGCTCGGTTTCCTTGTGGCGGTTCTGGCG
MAACWTPTCSARSHCGGSG	19	19	0	ATGGCAGCATGCTGGACGCCTACGTGCTCTGCTCGTTCGCATTGTGGCGGTTCTGGCG

Advanced options

- **Comparing only sequences whose abundance is higher than a certain cutoff (the same for all datasets):** specify the cutoff in the input as follows:
  CommonSeq('cutoff',n)
  Where "n" is the minimum abundance desired. For example, to compare only sequences whose abundance is higher than 10:

```
>> CommonSeq('cutoff',10)
```

- **Comparing only the most abundant clones from each dataset:** you can take, from each dataset, the most abundant sequences and compare them. For example, to compare the 100 most abundant sequences of each dataset:

```
>> CommonSeq('top',100)
Minimum abundance for dataset 1 = 3
File 1: considering 134 different sequences
File 1: considering 952 total sequences
Minimum abundance for dataset 2 = 3
File 2: considering 127 different sequences
File 2: considering 880 total sequences
Finished dataset 1
Finished dataset 2
```

→ If we take the 100 most abundant sequences, the minimum abundance we are considering is 3 in the case of dataset 1, and also 3 in the case of dataset 2.
It will take all sequences whose abundance is at least 3 and this can be higher than 100 (134 in the case of dataset 1 and 127 in the case of dataset 2)

- **Specifying a constant C-terminal peptide sequence to remove frame-shifted clones:** in order to not consider frame-shifted clones, a constant C-terminal peptide sequence can be indicated, so only peptides having it will be taken. It must be specified in the input as follows:
  `CommonSeq('cter','XYZ')`
  where XYZ is the constant C-terminal peptide sequence. For example, for bicyclic peptide libraries and the "start" and "end" of the peptide used by default, 'GGSG' can be used.

7. FindSeq

Function information

This script searches all the dataset for peptide sequences containing a specified motif, which must be indicated in the input. It can be a string of characters or a MatLab "regular expression" (see MatLab help for all the options). It distributes the peptides in two different files, according to whether they contain the specified motif or not.

Running the function

Type FindSeq('seq','XYZXYZ') in the command window:

```
>> FindSeq('seq','HPQ')
```

A dialog box will open allowing you to choose the file. The input files accepted are the output of Step2 (Translated....txt and fixerrTranslated....txt), and also the outputs of LoopLengths. The requisite is that the contents of the text file are in the format: peptide sequence – abundance - nucleotide sequence, e.g:

```
MAACTQSACSARVVCGGSG    76  ATGGCAGCATGCACGTAGTCTGCTTGCTCGGCGAGGGTTGTGTGTGGCGGTTCTGGCG
MAACAPDQCTKFTMCGGSG    34  ATGGCAGCATGCGCTCCGGATCAGTGCACTAAGTTTACTATGTGTGGCGGTTCTGGCG
MAACTYALCTARTFCGGSG    27  ATGGCAGCATGCACTTATGCTCTGTGCACTGCGCGTACGTTTTGTGGCGGTTCTGGCG
MAACSASQCSARIGCGGSG    21  ATGGCAGCATGCTCTGCTTCGTAGTGCTCTGCTAGGATTGGTTGTGGCGGTTCTGGCG
MAACKHSDCTARFPCGGSG    20  ATGGCAGCATGCAAGCATAGTGATTGCACTGCTCGGTTTCCTTGTGGCGGTTCTGGCG
MAACAQATSCQTARCGGSG    20  ATGGCAGCATGCGCGTAGGCGACTTCGTGCTAGACTGCGCGTTGTGGCGGTTCTGGCG
MAACWTPTCSARSHCGGSG    19  ATGGCAGCATGCTGGACGCCTACGTGCTCTGCTCGTTCGCATTGTGGCGGTTCTGGCG
MAACPVKPSCHSGRCGGSG    18  ATGGCAGCATGCCCTGTTAAGCCGTCTTGCCATTCTGGGAGGTGTGGCGGTTCTGGCG
MAACPVLPQCSARSCGGSG    18  ATGGCAGCATGCCCTGTGCTTCCTTAGTGCTCGGCGAGGTCTTGTGGCGGTTCTGGCG
MAACMLSGSCTARSCGGSG    17  ATGGCAGCATGCATGTTGTCTGGTTCTTGCACGGCTAGGTCGTGTGGCGGTTCTGGCG
```

Once running, the command window will display:

```
>> FindSeq('seq','HPQ')
Looking for motif: HPQ
considering all sequences
No minimum abundance specified
```

Advanced options (see below):

- Looking for motif: HPQ → Complex motifs can be indicated using MatLab regular expressions
- considering all sequences → A C-terminal constant peptide region can be indicated to remove frame-shifted clones
- No minimum abundance specified → A minimum abundance can be indicated

Output

Within the folder where the input file was, a new folder appears called "Seq", with the following files inside: Seq_XYZ_match.txt (containing peptide sequences having the motif), Seq_XYZ_nomatch.txt (containing peptide sequences without the motif), and Seq_XYZ_stats.txt (containing the number of sequences assigned to each file).

Advanced options

- **Searching for complex motifs:** see all the possibilities allowed by regular expressions in MatLab. For example, to look for HPXQ motif, where X is any amino acid, the expression is `'HP.Q'`; to look for $^{T}/_{S}$AR sequences, the expression is `'[TS]AR'`; to look for TAR and TR sequences, the expression is `'TA?R'`.

- **Specifying a constant C-terminal peptide sequence to remove frame-shifted clones:** in order to not consider frame-shifted clones, a constant C-terminal peptide sequence can be indicated, so only peptides having it will be taken. It must be specified in the input as follows:
  `FindSeq('seq','XYZ','cter','ZZZ')`
  where ZZZ is the constant C-terminal peptide sequence. For example, for bicyclic peptide libraries and the "start" and "end" of the peptide used by default, 'GGSG' can be used.

- **Specifying a minimum abundance cutoff:** it can be indicated in the input as follows:
  `FindSeq('seq','XYZ','cutoff',n)`
  where "n" is the minimum abundance for a peptide to be considered.
